# Does mHealth voice messaging work for improving knowledge and practice of maternal and newborn healthcare?

**DOI:** 10.1186/s12911-019-0903-z

**Published:** 2019-09-05

**Authors:** Mahbub Elahi Chowdhury, Shafayatul Islam Shiblee, Heidi E. Jones

**Affiliations:** 1 International Centre for Diarrhoeal Disease Research, Bangladesh (icddr,b), 68 Shaheed Tajuddin Ahmed Sarani, Mohakhali, Dhaka, 1212 Bangladesh; 2 CUNY Graduate School of Public Health & Health Policy, New York, USA

**Keywords:** Bangladesh, mHealth, Voice messaging, Maternal health, Neonatal health

## Abstract

**Background:**

*Aponjon* (meaning “near and dear ones”), a mobile phone-based mHealth service, customized voice messages for expectant (6–42 weeks pregnancy) and new mothers (1–52 weeks after delivery) for promotion of recommended healthcare practices. The *Aponjon* system sent two voice messages per week to subscribers, tailored to the timing during pregnancy or post-partum. The current study is an external evaluation of the effect of *Aponjon* use on knowledge and behaviors related to maternal and newborn health (MNH) care.

**Methods:**

We implemented an observational study of *Aponjon* users with propensity score matched non-users in Bangladesh. Subscribers with at least 3 months exposure to *Aponjon* and non-users were interviewed retrospectively on knowledge and practices surrounding MNH. The sample included women with infants ≤6 months (243 users; 369 non-user) for maternal health knowledge and practice indicators and women with infants > 6 to 12 months old (332 users; 454 non-user) for neonatal health knowledge and practice indicators. Data were analyzed using principal component analysis and categorized as ‘high’ and ‘low’ at the median of principal component scores. Interactions between duration of use of *Aponjon* services and self-reported patterns of receiving and listening to messages were examined to assess the effect on knowledge and practices for MNH.

**Results:**

Women reporting at least 6 months of using *Aponjon* were approximately 3 times as likely as the non-users to score high on both maternal healthcare knowledge questions and related practices. Similarly women with at least 6 months of *Aponjon* exposure were 1.5 times as likely as the non-users to score high on knowledge questions on newborn health. Reporting a good-pattern of *Aponjon* use (i.e. receiving a minimum of 3 messages per month and listening to all of them) had an even stronger association with knowledge and practices related to MNH care. However, a shorter exposure to Aponjon service (i.e. 3–5 months), despite having a good-pattern of use, did not have an effect on the related outcomes.

**Conclusions:**

The use of *Aponjon* services for at least 6 months, with a good-pattern of receiving and listening to the messages, was associated with improved knowledge and practices related to MNH care.

**Electronic supplementary material:**

The online version of this article (10.1186/s12911-019-0903-z) contains supplementary material, which is available to authorized users.

## Background

Extensive use of mobile telephones in many developing countries offers potential for the use of mHealth to disseminate pubilc health information [1]. Recent systematic reviews suggest that mHealth interventions can improve self-management of diabetes, weight loss, physical activity, quitting smoking, and adherence to medication in antiretroviral therapy [2, 3]. However, to date, few studies have evaluated whether mHealth can improve maternal and newborn health (MNH) among pregnant and post-partum women in low- or middle-income countries [4–9]. A randomized control trial of mHealth in Kenya found a significant increase in attendance of at least four antenatal care visits in the intervention group compared to the control group [4]. Another randomized controlled trial of a mobile phone intervention consisting of short messages combined with a health voucher in Zanzibar found a positive effect on both attending at least four antenatal care visits and using skilled delivery care [5, 6]. A controlled pre-post study in Thailand showed a significant increase in the proportion of children receiving timely vaccination after implementation of a mobile phone-based intervention with automated text message reminders for appointments [7]. A study in Nigeria, examining postnatal care attendance using historic controls from the previous 6 months in the same hospital, found that the intervention group that received text message appointment reminders was 50% more likely to attend their postnatal care appointments [8]. In terms of nutrition, a quasi-experimental study in Shanghai found that text messages about infant feeding resulted in a significantly higher rate of exclusive breastfeeding at 6 months and a significantly lower rate of the introduction of solid foods before 4 months [9].

Most of these studies assessed the effectiveness of mHealth on maternal and child health in facility settings and used text messages, which have limitations in settings with low literacy [4–9]. The current study aimed to test the effectiveness of an mHealth intervention--*Aponjon--*which used interactive voice messaging to improve knowledge of MNH care as well as increase following of recommended MNH practices in a large community setting in Bangladesh.

### Description of *Aponjon* mHealth service

*Aponjon*, a mobile phone-based mHealth service for expectant and new mothers, was developed and implemented by Dnet (a social franchise organization in Bangladesh) in collaboration with Global MAMA (Mobile Alliance for Maternal Action, USA). The goal of the *Aponjon* mHealth intervention was to disseminate behavior change communication messages quickly and easily to women about the need for prenatal care, dispelling myths and misconceptions around pregnancy and postpartum, building awareness of pregnancy and newborn warning signs, feeding practices for the newborn, postpartum family planning, and nutritional intake to improve MNH outcomes. Messages were tailored for two separate modules--one during pregnancy and another after delivery. The pregnancy-related module was designed for women during their 6–42 weeks of pregnancy and the module relating to recently delivered women was designed for new mothers, 1^_^52 week(s) post-partum.

To access the *Aponjon* services, clients had to subscribe to the service through a registration process by dialing a short code (16227). Dnet, the *Aponjon* service provider, collaborated with local non-governmental organizations whose health workers promoted *Aponjon* and assisted with the registration process. Once registered, subscribers received two Interactive Voice Response (IVR) messages per week, tailored to their point in pregnancy (based on self-reported date of last menstrual period or expected date of delivery) or the age of the child.

The *Aponjon* service was a commercial product and, thus, was not free of charge, except for subscribers from selected marginalized groups. For general subscribers, the service charge was BDT (Bangladeshi Taka) 2.00 per message; for subscribers with income below BDT 4000/month (half of the minimum wage) or education level below the 5th grade, the charge was BDT 1.00 per message; and, for subscribers from households headed by a female or a day-laborer, the service was provided free of charge. A subscriber could unsubscribe from the service any time, could listen to messages from previous weeks, and could listen to the same message repeatedly.

## Methods

We conducted a retrospective observational study of users and non-users of *Aponjon* mHealth service as part of an external evaluation; Dnet developed and implemented the *Aponjon* mHealth service independently. The study was conducted in four sites: (i) a rural surveillance area (Matlab) of International Centre for Diarrhoeal Disease Research, Bangladesh (icddr,b) which is about 110 km to the southeast of capital city Dhaka; (ii) a slum (Bhashantek) in Dhaka; (iii) an urban area of a typical district (Brahmanbaria) which is about 111.4 km to the northeast of Dhaka; and (iv) a rural area in the same district. These sites were chosen purposively. Icddrb's Matlab site was selected as an existing surveillance system facilitated participant selection. We selected the urban slum to test the *Aponjon* intervention in a population of low socioeconomic status in a metropolitan area. We selected the urban and rural areas of Brahmanbaria to test how the intervention worked in the general population. The study was approved by the Institutional Review Board of icddr,b.

In each study area, two samples of women were included. Mothers with infants ≤6 months of age were interviewed to assess the effect of *Aponjon* on knowledge and practice for maternal healthcare during pregnancy and those within > 6 to 12 month old infants were examined for the effect of the intervention on knowledge and practice of newborn healthcare during the infants’ first 6 months of age. Based on the hypothesis that a sufficient threshold of use would be needed to have an effect, we enrolled women with at least 3 months of use of *Aponjon services,* either during pregnancy (for maternal health outcomes) or within the first 6 months of the post-partum period (for newborn health outcomes).

### Data-collection process

The selection of participants for both sample populations was done in four phases. In Phase I, we identified areas (blocks in urban areas and villages in rural areas) with a minimum *Aponjon* subscription of 1% of urban households (Bhashantek slum and urban Brahmanbaria) and 2% of rural households. Based on these criteria, one slum in Bhashantek (4020 households) and 24 blocks in urban Brahmanbaria (15,852 households), 7 villages in Matlab (3271 households), and 98 villages in rural Brahmanbaria (43,235 households) were selected.

**Fig. 1:**
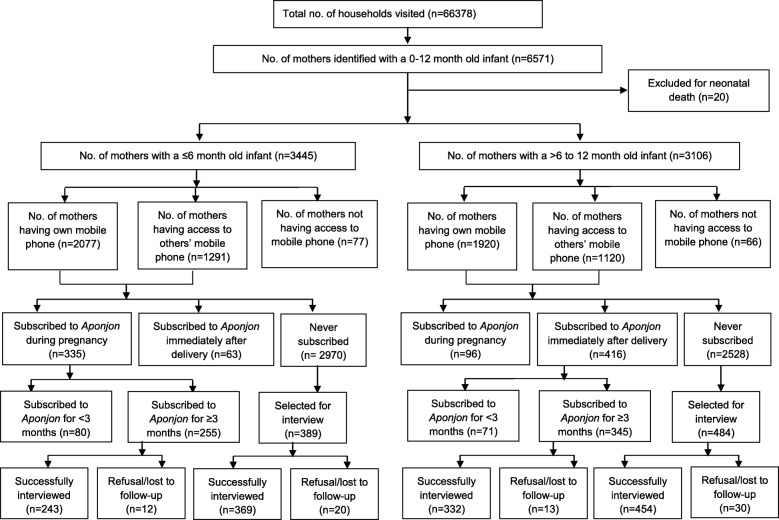
CONSORT flow diagram for selection of study subjects

In Phase II, all households (*n* = 66,378) in the selected areas were visited to identify women with children below 1 year of age and with access to a mobile phone. Accessibility to mobile phone was defined as either owning a mobile phone or having access to a phone of another household member. Women who experienced neonatal death recently or did not have access to a mobile phone were excluded. From visiting 66,378 households, the study identified 3445 mothers with an infant ≤6 months and 3106 mothers with a child > 6 to 12 months of age. Among mothers with infants ≤6 months of age, we identified 255 who had used *Aponjon* for at least 3 months during pregnancy and 2970 mothers who had never used it during pregnancy. Similarly, among mothers with > 6 to 12 month old infants, we identified 345 mothers who had used *Aponjon* for at least 3 months during the first 6 months post-partum and 2528 mothers who had never used *Aponjon* during that period (Fig. 1).

In Phase III, we selected all of the mothers identified who had used Aponjon and matched them to two controls from the never users, using propensity score matching (PSM). Propensity scores were estimated based on mother’s age, mother’s education, father’s education, and household wealth index within each of the four sites. Initially, for each exposed participant, two non-exposed participants were selected by applying the nearest neighborhood matching method [10]. Controls selected more than once due to proximity of the propensity score with more than one exposed participant were included only once, resulting in 389 matched controls for the 255 *Aponjon* users during pregnancy and 484 matched controls for the 345 *Aponjon* users during the post-partum period.

In Phase IV, all selected participants were re-visited for interviews using pretested structured questionnaires designed to cover the topics covered in the *Aponjon* messages. Mothers with ≤6 month old infants were asked questions about their knowledge and recommended practices relating to maternal healthcare during the last pregnancy and around the time of delivery. Mothers with > 6 to 12 month old infants were asked about their knowledge and practices regarding newborn healthcare around the time of delivery through 6 months post-partum. The mothers who had subscribed to *Aponjon* were also asked about the extent to which they received and listened to the messages. The questionnaires were administered by a team of 32 trained female interviewers divided into four teams under the guidance of four supervisors. Prior to data collection, the full team was intensively trained for 2 weeks on the data-collection tools, including interview techniques. The study investigators closely monitored the data-collection process in the field by making surprise field visits and on-the-spot checks of a sample of completed questionnaires. All completed questionnaires were checked for internal validity.

### Data analysis

We estimated the effect of being exposed to *Aponjon* services on four different outcome domains, based on the messages of the services: (i) knowledge of maternal healthcare (22 indicators); (ii) behaviors relating to maternal healthcare (19 indicators); (iii) knowledge of newborn healthcare (23 indicators); and (iv) behaviors relating to newborn health (12 indicators; see Additional file 1 for full questionnaire). For each domain, a composite dichotomous outcome measure was created using principal component analysis with orthogonal rotation of related indicators. We dichotomized the principal component score for each domain at the median. We also assessed the dose-response effect by examining the association of outcome variables with the ordinal exposure variable at 0, 3–5 and ≥ 6 months of use of *Aponjon* services. We further tested for interaction between duration of use of *Aponjon* services and a dichotomous measure of ‘pattern of receiving and listening to the messages’. We defined this pattern as a “good pattern” for women who self-reported having received at least 3 messages per month and carefully listening to most of the messages or as a “poor pattern” for women who reported having received fewer than 3 messages per month and/or not listening to most of the messages carefully. For each domain, we present bivariate and multivariable logistic regression, adjusting for a priori hypothesized confounders not included in the propensity score (ownership of mobile phone as an indicator of socioeconomic position, rural vs. urban residence, and exposure to other forms of media) as well as clustering at the block/village levels. We used a critical alpha value of 0.05 to determine statistical significance.

## Results

Of the 6551 mothers with infants under 1 year old, 61.0% (3997/6551) had their own mobile phone, 36.8% (2411/6551) had access to other’s phones, and 2.2% (143/6551) did not have any access to a mobile phone (Fig. 1). Overall, among mothers with infants ≤6 months of age with access to a mobile phone, only 9.9% (335/3368) subscribed to *Aponjon* during pregnancy and 7.6% (255/3368) used the service for at least 3 months. Among mothers with infants > 6 to 12 months old, only 13.7% (416/3040) subscribed to *Aponjon* within the first 6 months of delivery, and 11.3% (345/3040) used the service for at least 3 months during that period.

Response rates in all groups were high: 95.3% (243/255) of mothers who used *Aponjon* services during pregnancy and 96.2% (332/345) of mothers who used *Aponjon* post partum were successfully interviewed. The rate of successful interviews in the respective control groups were 94.9% (369/389) and 93.8% (454/484) respectively (Fig. 1).

As shown in Table 1, about four-fifths of the participants lived in rural areas and one-fifth in an urban area. The slum represented only about 1.0% of participants. The majority of participants in each group were between 20 and 29 years of age, had at least primary education, belonged to the top two socioeconomic quintiles, and had exposure to at least one form of mass media (Table 1). After PSM, the background characteristics of the participants were similar between the intervention and control groups for both categories of women (with infants ≤6 months and > 6 to 12 months), except for ownership of mobile phones. In both groups, a relatively higher proportion of *Aponjon* service-users owned a mobile phone compared to non-users.

**Table 1:** Comparison of background characteristics between the propensity score matched users and non-users of *Aponjon* services

Characteristics	Mothers of infants ≤6 months of age			Mothers of infants > 6 to12 months of age		
	Number of users (%)	Number of non users (%)	*p*-value	Number of users (%)	Number of non users (%)	*p*-value
Study areas						
Bhasantek slum in Dhaka	2 (0.8%)	4 (1.1%)	0.48	4 (1.2%)	4 (0.9%)	0.18
Urban Brahmanbaria	33 (13.6%)	67 (18.2%)		40 (12.1%)	80 (17.6%)	
Rural Brahmanbaria	201 (82.7%)	287 (77.8%)		283 (85.2%)	362 (79.7%)	
Matlab field site of icddr,b	7 (2.9%)	11 (2.9%)		5 (1.5%)	8 (1.8%)	
Mother’s age						
< 19 years	35 (14.4%)	57 (15.5%)	0.98	41 (12.4%)	65 (14.3%)	0.63
20–24 years	84 (34.6%)	125 (33.9%)		122 (36.8%)	169 (37.2%)	
25–29 years	73 (30.0%)	113 (30.6%)		108 (32.5%)	130 (28.6%)	
30 years and above	51 (21.0%)	74 (20.1%)		61 (18.4%)	90 (19.8%)	
Mother’s education						
Incomplete primary	35 (14.4%)	55 (14.9%)	0.98	40 (12.1%)	64 (14.1%)	0.55
Primary	69 (28.4%)	109 (29.5%)		105 (31.6%)	153 (33.7%)	
Secondary	86 (35.4%)	128 (34.7%)		113 (34.0%)	152 (33.5%)	
Higher	53 (21.8%)	77 (20.9%)		74 (22.3%)	85 (18.7%)	
Father’s education						
Incomplete primary	62 (25.5%)	103 (27.9%)	0.89	71 (21.4%)	96 (21.2%)	0.99
Primary	54 (22.2%)	75 (20.3%)		105 (31.6%)	149 (32.8%)	
Secondary	75 (30.9%)	111 (30.1%)		79 (23.8%)	105 (23.1%)	
Higher	52 (21.4%)	80 (21.7%)		77 (23.2%)	104 (22.9%)	
Household asset quintiles						
Lowest	26 (10.7%)	39 (10.6%)	0.56	26 (7.8%)	46 (10.1%)	0.48
Second	42 (17.3%)	68 (18.4%)		65 (19.6%)	87 (19.2%)	
Middle	49 (20.2%)	93 (25.2%)		69 (20.8%)	87 (19.2%)	
Fourth	58 (23.9%)	82 (22.2%)		83 (25.0%)	130 (28.6%)	
Highest	68 (27.9%)	87 (23.6%)		89 (26.8%)	104 (22.9%)	
Ownership of mobile phone						
Used others’ phone	74 (30.5%)	141 (38.2%)	0.05	81 (24.4%)	173 (38.1%)	0.00
Had own phone	169 (69.6%)	228 (61.8%)		251 (75.6%)	281 (61.9%)	
Exposure to mass media						
No exposure	76 (31.3%)	126 (34.15%)	0.65	100 (30.1%)	162 (35.7%)	0.26
Exposure to 1 form of media	133 (54.7%)	199 (53.9%)		187 (56.3%)	234 (51.5%)	
Exposure to ≥2 forms of media	34 (13.9%)	44 (11.9%)		45 (13.6%)	58 (12.8%)	

### Use of Aponjon services

Not all *Aponjon* service-users received all 8 messages per month as sent by *Aponjon*. Overall, 84.3% (485/575) of users said that they had received at least 3 messages per month. Of them 54.4 and 29.9% had received 3–5 and 6–8 messages per month respectively. The remaining 15.7% (90/575) reported receiving only 1–2 messages per month. Three major reasons cited for not receiving the phone call were due to household chores (72%), someone else owning the mobile phone (48%), and airtime cost for *Aponjon* services (10%). Upon receiving the call, not all women carefully listened to the messages. Only about 35.5% (204/575) women said that they had carefully listened to all of the messages they had receivedand the rest 64.5% (371/575) listened to only some of the messages. Thirty-two percent (184/575) of *Aponjon* subscribers who had received at least 3 messages per month and listened to all the messages carefully were defined as having a ‘good pattern of receiving and listening to the messages’. The remaining 68.0% (391/575) who had either received < 3 messages per month or not carefully listened to all of the messages were defined as having a ‘poor pattern of receiving and listening to the messages’.

### Effect of Aponjon on individual knowledge and behavior indicators

Table 2 demonstrates the crude association of the use of *Aponjon* services with each of the indicators in the four domains. For maternal health knowledge, 7 (41%) indicators (correct timing of seeking 4 different antenatal cares (ANCs), place of ANC, importance of blood grouping, symptoms of eclampsia, pregnancy danger signs, place of seeking postnatal care (PNC), and duration of birth spacing) out of 22 were higher among *Aponjon* service-users. For behaviors relating to maternal healthcare, 8 (42%) indicators (seeking ANC from skilled care providers, number of ANC visits made, receiving ANC at the recommended gestational period, number of diagnostic tests done, taking initiative for different birth preparedness components, taking iron-folic acid during pregnancy, taking vitamin A capsules after delivery, taking vitamin A capsules within 1–7 day(s) of delivery) out of 19, were significantly associated with the use of *Aponjon* services. Similarly, for the knowledge of newborn healthcare, 7 out of 23 (30%) indicators (knowledge on symptoms of infected umbilical cord, correctly reporting on days of delayed bathing of the newborn, benefits of feeding colostrum, benefits of exclusive breastfeeding, understanding if sufficient breast-milk had been sucked by the baby, types of diseases preventable by immunization, and time of seeking care for diarrhea of the baby) were positively associated with the use of *Aponjon* services. In addition, in each of these three domains, a number of indicators (responses on reasons for Tetanus Toxoid vaccination, correct reporting on recommended lying position during pregnancy, responses on birth preparedness components, correct reporting on recommended number (four) of postpartum care visits, receiving care from facilities for complications during pregnancy, responses about the place of vaccination, responses on type of care to be given for diarrhea) were also of borderline significance (*p* < 0.10). Surprisingly, for behaviors relating to newborn healthcare, none of the 12 indicators studied were different between users of *Aponjon* services and non-users.

**Table 2:**
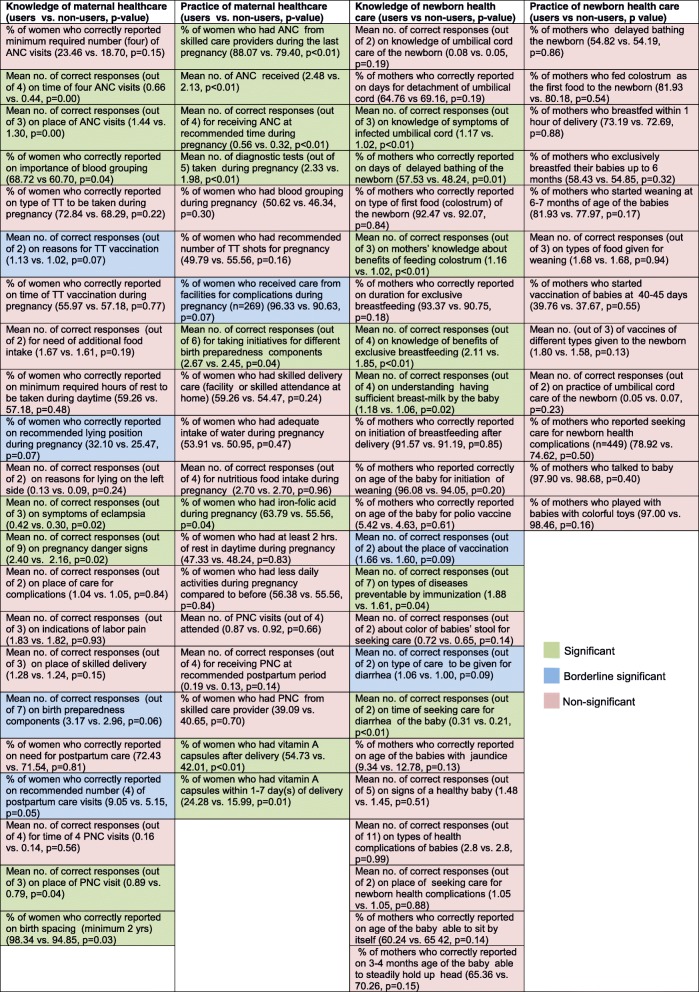
Variations in outcome indicators of knowledge and practices for maternal and newborn health for users and non users of *Aponjon* services

### Effect of Aponjon on composite knowledge and behavior measures

As seen in Table 3, after adjustment of hypothesized confounders, 3–5 months of the use of *Aponjon* services was not associated with increased maternal healthcare knowledge as a composite score (Adjusted OR, aOR: 1.20, 95% CI 0.80, 1.81) or practices (aOR: 1.18, 95% CI 0.81, 1.72) compared to non-users. However, at least 6 months of *Aponjon* use was associated with a 3 times higher likelihood of a high maternal healthcare knowledge score (aOR = 2.83, 95% CI 1.57, 5.09) and recommended related practices (aOR = 3.38, 95% CI 1.77, 6.48). Further, women who reported a ‘good pattern of receiving and listening to messages’ were about twice as likely to have a higher knowledge score (aOR = 2.00, 95% CI 1.17, 3.37) and related practices (aOR = 1.96, 95% CI 1.09, 3.51) for maternal healthcare compared to non-users.

**Table 3:** Crude and adjusted odds ratios (ORs) for increased knowledge of mothers and related practices for maternal healthcare by exposure to *Aponjon* service and its use pattern

*Aponjon* exposure and use pattern	*n* = 612	Knowledge of maternal healthcare			Practice of maternal health care		
		% scored ≥50th percentile	Crude OR^ (95% CI)	Adjusted OR^a^ (95% CI)	% scored ≥50th percentile	Crude OR^ (95% CI)	Adjusted OR^a^ (95% CI)
Exposure to *Aponjon*							
No exposure (non-user)	369	45.3	1.00	1.00	45.0	1.00	1.00
3–5 months exposure	159	49.7	1.19 (0.82–1.73)	1.20 (0.80–1.81)	48.4	1.15 (0.79–1.67)	1.18 (0.81–1.72)
6–9 months exposure	84	71.4	3.02 (1.80–5.07)	2.83 (1.57–5.09)	75.0	3.67 (2.15–6.26)	3.38 (1.77–6.48)
Use pattern of Aponjon							
No exposure (non-user)	369	45.3	1.00	1.00	45.0	1.00	1.00
Did not receive or listen to ≥3 messages per month (poor pattern)	169	53.9	1.14 (0.98–2.03)	1.45 (0.92–2.29)	54.4	1.46 (1.01–2.10)	1.54 (1.03–2.29)
Received and listened to ≥3 messages per month (good pattern)	74	64.9	2.23 (1.33–3.75)	2.00 (1.17–3.37)	64.9	2.26 (1.34–3.80)	1.96 (1.09–3.51)
Exposure to Aponjon and use pattern							
No exposure (non-users)	369	45.3	1.00	1.00	45.0	1.00	1.00
Subscribed for 3–5 months and had a poor pattern of use	112	45.5	1.01 (0.66–1.55)	1.07 (0.65–1.76)	43.8	0.95 (0.62–1.46)	1.03 (0.67–1.59)
Subscribed for 3–5 months and had a good pattern of use	47	59.6	1.78 (0.96–3.31)	1.62 (0.87–3.01)	59.6	1.80 (0.97–3.34)	1.67 (0.81–3.43)
Subscribed for 6–9 months and had a poor pattern of use	57	70.2	2.85 (1.56–5.20)	2.77 (1.41–5.44)	75.4	3.76 (1.99–7.10)	3.79 (1.88–7.66)
Subscribed for 6–9 months and had a good pattern of use	27	74.1	3.46 (1.43–8.37)	2.97 (1.09–8.09)	74.1	3.49 (1.44–8.46)	2.66 (0.92–7.62)

^a^Adjusted for area**,** ownership of mobile phone, exposure to mass media, cluster; ^based on propensity score matched sample

When we further examined the duration of use with the ‘pattern of receiving and listening to messages’ of *Aponjon*, 3–5 months of use of *Aponjon* services, despite having a ‘good pattern of receiving and listening to messages’ had no effect on knowledge and practice surrounding maternal healthcare. However, at least 6 months of *Aponjon* service-use with ‘good pattern of receiving and listening to messages’ increased the odds of high knowledge by about 3 times (aOR = 2.97, 95% CI 1.09, 8.09). as well as practices (aOR = 2.66, 95% CI 0.92, 7.62, Table 3).

As seen in Table 4, our analysis on the effect of *Aponjon* services on newborn healthcare showed that 3–5 months of use was not associated with increased knowledge (aOR = 1.19, 95% CI 0.67, 2.12), while use for ≥6 months was associated with increased knowledge (aOR = 1.46, 95% CI 1.05, 2.03). Duration of use of *Aponjon* services was not associated with newborn practices. However, at least 6 months of use with a ‘good pattern of receiving and listening to messages’ was associated with higher scores for both knowledge (aOR = 1.63, 95% CI 1.02, 2.61) and practices (aOR = 1.63, 95% CI 1.06, 2.51) related to newborn healthcare.

**Table 4:** Crude and adjusted odds ratios (ORs) for increased knowledge of mothers and related practices for newborn healthcare by exposure to *Aponjon and its use pattern*

*Aponjon* exposure *and use pattern*	*n* = 786	Knowledge for newborn health care			Practice of newborn health care		
		% scored ≥50th percentile	Crude OR^ (95% CI)	Adjusted OR^a^ (95% CI)	% scored ≥50th percentile	Crude OR^ (95% CI)	Adjusted OR^a^ (95% CI)
Exposure to *Aponjon*							
No exposure (non-user)	454	45.6	1.00	1.00	48.5	1.00	1.00
3–5 months exposure	51	51.0	1.24 (0.70–2.21)	1.19 (0.67–2.12)	41.2	0.74 (0.41–1.34)	0.77 (0.42–1.40)
6–12 months exposure	281	56.9	1.58 (1.17–2.13)	1.46 (1.05–2.03)	54.1	1.25 (0.93–1.69)	1.22 (0.91–1.64)
Use pattern of Aponjon							
No exposure (non-user)	454	45.6	1.00	1.00	48.5	1.00	1.00
Did not receive or listen to ≥3 messages per month (poor pattern)	222	52.7	1.33 (0.96–1.83)	1.28 (0.91–1.80)	47.3	0.95 (0.69–1.32)	0.96 (0.71–1.31)
Received and listened to ≥3 messages per month (good pattern)	110	62.7	2.01 (1.31–3.08)	1.76 (1.10–2.80)	61.8	1.72 (1.12–2.64)	1.63 (1.04–2.55)
Exposure to *Aponjon* and use pattern							
No exposure (non-users)	454	45.6	1.00	1.00	48.5	1.00	1.00
Subscribed for 3–5 months and had a poor pattern of use	41	43.9	0.93 (0.49–1.78)	0.91 (0.47–1.77)	36.6	0.61 (0.32–1.19)	0.64 (0.35–1.16)
Subscribed for 3–5 months and had a good pattern of use	10	80.0	4.77 (1.00–22.72)	4.30 (1.12–16.52)	60.0	1.60 (0.44–5.73)	1.65 (0.39–6.97)
Subscribed for 6–9 months and had a poor patter of use	181	54.7	1.44 (1.02–2.04)	1.38 (0.96–1.99)	49.7	1.05 (0.75–1.48)	1.06 (0.75–1.48)
Subscribed for 6–9 months and had a good patter of use	100	61.0	1.87 (1.20–2.90)	1.63 (1.02–2.61)	62.0	1.74 (1.11–2.70)	1.63 (1.06–2.51)

^a^Adjusted for area**,** ownership of mobile phone, exposure to mass media, cluster; ^based on propensity score matched sample

## Discussion

Almost all women either owned a personal cell phone (61%), and/or had access to a household member’s phone (37%). However, in the study areas, only about 8 and 11% of the respondents used *Aponjon* for at least 3 months during pregnancy and early motherhood, respectively. Our study demonstrates that subscribers to the *Aponjon* mHealth intervention had higher knowledge and positive self-reported behaviors for improved maternal health than non-subscribers. Mothers who subscribed to *Aponjon* for at least 6 months and reported receiving and listening to messages had better knowledge and reported more recommended behaviors for both maternal and newborn healthcare compared to non-users.

Most previous studies that have examined the impact of mHealth services have focused on selected components of MNH care, like ANC [4, 5, 7], PNC, and immunization coverage [8]. All of these studies were facility-based and, thus, might not be generalizable to community settings. Moreover, all of these studies used text messages to remind mothers of an upcoming routine check-up or vaccination for their children. Conversely, we, for the first time, examined the effectiveness of a package of voice messages to increase knowledge and promote good practices in four different domains of MNH care in community settings. We also examined how using the mHealth service for an extended period and having a good pattern of use impacted its effect.

Our findings showed that exposure to *Aponjon* was associated with better knowledge of mothers about when and where to access ANC services. This finding is consistent with recent studies which found that mHealth services increased antenatal care attendance [4, 6, 11].

Our study also demonstrated that *Aponjon* can increase knowledge on the symptoms of pregnancy-related complications. We found greater knowledge about signs of eclampsia and initiatives to be taken for different birth preparedness components among *Aponjon* service-users compared to non-users. Thus, mHealth interventions have the potential to sensitize women about the second major cause of maternal death and promote good practices around the time of delivery, which is critical for saving the lives of the mother and her newborn.

PNC attendance, which begins 1 h after the delivery through 6 weeks post-partum [12], promotes healthy behaviors, danger sign recognition, and family planning practices. PNC also promotes extra care needed for low-birth-weight babies or babies born to HIV-positive mothers and babies requiring other specialized care [12]. Missing a PNC attendance contributes to maternal morbidity and mortality [8]. In our study, *Aponjon* increased knowledge of where to seek PNC, although it did not increase PNC attendance.

Vitamin A deficiency is a significant public health problem in Africa and South-East Asia, as it can cause Nyctalopia and may increase the risk of death and sickness from childhood viral infections, like measles [13]. In our study, we found 24% of *Aponjon* service-users, compared to 16% of non-users, had taken vitamin A within 7 days of delivery, and the difference was statistically significant. While this is an improvement, as the majority of women in both the groups did not report taking vitamin A, other interventions are also needed.

In our study, none of the individual indicators around behaviors for newborn care was associated with the use of *Aponjon* services. However, we did see some improvement in behavior around newborn care among the *Aponjon* users who subscribed to the service for more than 3 months and had a good pattern of receiving and listening to messages.

Only about 8 and 11% of our study population used *Aponjon* for at least 3 months during pregnancy and after delivery respectively. There was also substantial variation in the subscription to *Aponjon* among the four study areas. Relatively higher subscription rates in rural Brahmanbaria district was due to strong networking with implementation partners for promotion of *Aponjon* and a relatively longer duration of the intervention in that area. The challenge that the *Aponjon* program faced was a low subscription rate in the slum areas. A complementary qualitative study confirmed that health workers tended to choose relatively educated women from well-off families for the subscription to avoid concerns regarding the costs of the service [14]. Innovative programmatic efforts are needed for increasing the subscription rates in slum areas. To bring more poor women under the network, the program should rethink further enhancing the current pricing system (for subscribers with income below BDT 4000 or education level below the 5th grade, it costs BDT 1.00 per message/min; and, for subscribers from households headed by female or day-laborer the service is provided free of charge) for a safety-net program. The selection process of the clients for discounted pricing also should be reviewed to ensure that poor clients can benefit.

Missing the calls and/or not listening to messages carefully detracted from the positive effect of the intervention. As women reported not being able to listen due to other household obligations, initiatives should be taken by *Aponjon* to determine the best time of day to send messages, perhaps allowing users to select preferred time for receipt of messages to allow for different work/household schedules. Further, some incentivized mechanism could be built into the *Aponjon* package, such as discounted call rates for attaining a high score in knowledge tests to increase the motivation to listen to all messages carefully. For increased accessibility, the program could consider offering low-cost or used phones to individuals who do not own phones. By the end of November 2018, in Bangladesh, the total number of mobile phone subscriptions had reached 157 million, and this number is increasing daily [15]. Accessibility of mobile phones among new mothers in our study was also quite high. About 61% of the mothers owned a mobile phone, and nearly 37% had access to other’s phones. These figures demonstrate the huge potential for using mobile phone for awareness raising for MNH in Bangladesh. On the other hand, Bangladesh is one of 57 countries with a severe shortage of trained doctors, paramedics, nurses, and midwives, [16] and any mHealth interventions must be combined with strengthening the presence of trained providers.

As the use of cell phones is increasing rapidly, there is an opportunity to improve health outcomes through the use of mHealth. The Bangladeshi Government has a mandate to establish ‘Digital Bangladesh’, and mHealth is a priority for health service delivery [17]. It is also worthwhile exploring the benefits of mHealth technology in reducing maternal and neonatal morbidity and morbidity. For about a quarter of the population of the country living below poverty level, who no longer consider mobile phones a luxury item [17], mHealth may be a mechanism to improve MNH outcomes.

### Limitations of the study

This study had a number of limitations. Our outcome measures were knowledge and self-reported behaviors, which can be biased measures. Further, while we adjusted for hypothesized confounders through the use of PSM and multivariable regression, our results may be biased with residual confounding. Women who maintained their *Aponjon* subscription for 3 or more months may have had higher baseline knowledge prior to the subscription; however, the fact that listening to the messages carefully was associated with higher knowledge suggests that the messages themselves may have contributed to improved knowledge and practice. Finally, we were not able to test for the effect of exposure to *Aponjon* services in urban and rural areas separately.

## Conclusions

Our research found that using Aponjon services for at least 6 months during pregnancy was associated with increased knowledge and positive behaviors during pregnancy, as well as knowledge of newborn healthcare after delivery. Overall, reporting a good pattern of using Aponjon services (receiving a minimum of 3 messages per month and listening to all messages carefully) was associated with increased knowledge as well as practices for both maternal and newborn health. A shorter exposure (i.e. 3–5 months), despite having a good use pattern of Aponjon service was not associated with these outcomes. Reasons for not receiving and carefully listening to all of the messages should be further explored and, accordingly, programmatic initiatives should be taken to overcome these problems for greater benefit of the intervention. Furthermore, future research should assess the impact of the use of *Aponjon* services on MNH outcomes, rather than knowledge and practice.

## Additional file

Additional file 1: Study questionnaires. (ZIP 380 kb)

## Data Availability

The detailed dataset is available with MEC, the Principal Investigator of the main study. A copy of the original data is also stored in the data archive of icddr,b. These data are not publicly available. However, non-identifiable data can be accessed upon request subject to approval of the Research Administration Department of icddr,b.

## Abbreviations

ANC: Antenatal Care
CI: Confidence Interval
MNH: Maternal and Newborn Health
OR: Odds Ratio
PNC: Postnatal Care
PSM: Propensity Score Matching
