# Supplementary material for: Does mHealth voice messaging work for improving knowledge and practice of maternal and newborn healthcare?
Source: BMC Med Inform Decis Mak. 2019 Sep 5;19:179. doi: 10.1186/s12911-019-0903-z (PMC6727538; doi:10.1186/s12911-019-0903-z)
Supplement: Supplementary file 1 — Study questionnaires. (ZIP 380 kb) [file 12911_2019_903_MOESM1_ESM.zip › Questionnaire for mothers with 7-12 month old babiesR3.docx]

**Survey questionnaire with consent form for mothers with 7-12 months old babies; for user and non-user of Aponjon mobile services**

**Protocol Title:**

**Title: Accessibility, acceptability and effectiveness of Aponjon mobile phone based health information messages for behaviour change for improved health care practices: a study in four selected areas of Bangladesh”**

Protocol number: 12052

**ID no**. |__|__|__|__|__|__| Household sl no. |__|__|__|__|__|__|

| **Group name:** | **Aponjon user** = 1  **Aponjon non-user** = 2 | | \|__\| |
| --- | --- | --- | --- |
| **Study area :** | **Brahmanbaria rural** = 1  **Brahmanbaria urban** = 2  **Bhashantek slum** = 3  **Matlab icddr,b control area** = 4 | | \|__\| |
|  | | | |
| Respondent’s name: | | _ _ _ _ _ _ _ _ _ _ _ _ _ _ _ _ _ _ _ __ _ _ _ _ _ _ _ _ _ _ _ _ _ _ _ _ _ | |
| Responden’t age (years) | | _ _ _ _ _ _ _ _ _ _ _ _ _ _ _ _ _ _ _ _ _ _ _ _ _ _ _ _ _ _ _ _ _ _ _ _ _ | |
| Name of household head: | | _ _ _ _ _ _ _ _ _ _ _ _ _ _ _ _ _ _ _ _ _ _ _ _ _ _ _ _ _ _ _ _ _ _ _ _ _ _ | |
| Address : | | _ _ _ _ _ _ _ _ _ _ _ _ _ _ _ _ _ _ _ _ _ _ _ _ _ _ _ _ _ _ _ _ _ _ _ _ _ _  _ _ _ _ _ _ _ _ _ _ _ _ _ _ _ _ _ _ _ _ _ _ _ _ _ _ _ _ _ _ _ _ _ _ _ _ _ _ | |

| **Information of interviewer** | | | | |
| --- | --- | --- | --- | --- |
|  | 1^st^ Visit | 2^nd^ Visit | 3^rd^ Visit |  |
| Date: | _ _ / _ _ /_ __ _ | _ _ / _ _ /_ __ _ | _ _ / _ _ /_ __ _ |  |
| Name of interviewer |  |  |  |  |
| Date of Next interview (If any) |  |  |  |  |
| Time of Next interview (If any) |  |  |  |  |
| Result of interview* |  |  |  | \|__\| |

| - **Final result of interview** | Complete= 1 | \|__\| |
| --- | --- | --- |
|  | Partial=2 |  |
|  | Refuse=3 |  |
|  | Others (Specify)_ _ _ _ _ _ _ _ _ _ _ _ _ _=4 |  |
|  |  | |

**Time of interview start:** |__|__| : |__|__| (Railway time) Date |__||__| |__||__| |__||__||__||__|

**Consent for survey questionnaire for mothers with 7-12 months old babies; for user and non-user of Aponjon mobile services**

**Protocol Number: PR- 12052**

Protocol Title: **“Accessibility, acceptability and effectiveness of Aponjon mobile phone based health information messages for behaviour change for improved health care practices: a study in four selected areas of Bangladesh”**

**Principle Investigator’s name: Dr. Mahbub Elahi Chowdhury**

**Organization: ICDDR, B: Centre for Equity and Health Systems**

**Purpose of the Research Project:**

My name is ………….............. . We work in the International Centre for Diarrheal Disease Research, Bangladesh (ICDDR, B), known as the Cholera Hospital in Dhaka where researches on different types of health issues are conducted. We are here to know about the mHealth programme on Mobile Alliance for maternal action (MAMA)/ Aponjon. We are seeking your permission to include you as a participant in this study. The objective of this study is to explore what people know about this mHealth programme, what health services they are receiving through this programme, what constraints they are facing for availing the services. Also, we would like to know how much you know about maternal and child health and how are you solving your health problems with/ without the support of various messages that you receive from different sources.

**Why you are invited to participate in the study?**

You have been asked to participate in this study because you are a mother who is using/not using the mobile based health services called Aponjon. If you agree to participate in this study we will ask you about your experiences, feelings, attitudes, and notions regarding this service. More specifically, we will seek to understand more from you regarding the maternal and child health information services through mobile phone or other sources. We would also like to know about the involvement of your husband, other family members and neighbours regarding this.

The data collection time may take from 45 minutes to 1 hour. But if you do not wish to spend all of that time at once, we can always come back at a later time. We would like to fill out a questionnaire form in order to note down the valuable information you give. The information you will provide will solely be used for this particular study and nobody besides the research investigators will be able to access it.

**Risks and benefits:**

There are no risks to you, or your baby from taking part in the study. Any information that you answer or regret to answer will have absolutely no effect on the health services you are receiving during pregnancy/delivery/post natal period or general healthcare from the nearest health facility. If you have any resentment regarding our study, then please note the contact information below and you can register your complain there.

With the help of the information you will provide by participating in this study, we will be able to understand how much beneficial the maternal and newborn health related messages that are being sent by the mHealth programme are to the people. This study will help in future in establishing mHealth policy in sending health messages to mothers of newborns. As a result maternal and newborn health will be improved.

**Privacy, anonymity and confidentiality:**

Your identity will remain confidential. Only investigators of this study will have access to study forms which will be stored in locked cabinets at our head office in Dhaka.

**Future use of information:**

Only anonymous information will be used from this interview for the objectives described to you a few moments ago. We will never use your name or your contact information in any way. Instead, we will be using an identification number so that no one can identify you.

**Right not to participate and withdraw:**

Your participation in this study is completely voluntary. You can refuse to participate in the study any time. If you don’t want to participate or if you do not wish to answer any question, you are free to refuse it.

Once you agree to participate after completely understanding the purpose of this study and your role in this project, you will be asked to sign the consent form. If you cannot sign your name, we will be requesting you to give thumbprint impression on the form. By signing the consent form you indicate that you understand the information provided here and that you give your consent to participate in this research project.

**Principle of compensation:**

Your participation is completely voluntary and you will not get any financial benefit from participating in the study. Dr. Mahbub Elahi Chowdhury is the Principle Investigator of this study. If you have any question about this study, you may contact the persons listed in the following:

**For further query please contact:**

| **Dr. Mahbub-E-Elahi Chowdhury Khan**  Scientist, Centre for Equity and health Systems,  ICDDR,B, Mohakhali, Dhaka 1212,  Phone: 9827001-10/2215  Mobile: 01713090869  E-mail: melahi@icddrb.org | M A Salam Khan  Committee coordination secretariat  ICDDR,B; Mohakhali; Dhaka 1212  Phone: 9827001-10/3206  Mobile: 01711-428989  E-mail: salamk@icddrb.org |
| --- | --- |

Do you have any questions? Yes No

Do you agree to participate in this research project? Yes No

Name of participants:_______________________________

Signature/thump impression of the participants: ___________________________________

Name of person obtaining consent: ___________________________________

Signature of Person Obtaining Consent____________________ Date______________

Signature of Person as witness of Obtaining Consent ____________Date______________

**Assesnt for survey questionnaire for mothers with 7-12 months old babies; for user and non-user of Aponjon mobile services**

**Protocol Number: PR- 12052**

Protocol Title: **“Accessibility, acceptability and effectiveness of Aponjon mobile phone based health information messages for behaviour change for improved health care practices: a study in four selected areas of Bangladesh”**

**Principle Investigator’s name: Dr. Mahbub Elahi Chowdhury**

**Organization: ICDDR, B: Centre for Equity and Health Systems**

**Request from the guardians for interviewing women age 11-17 years old:** My name is …………We are working with the International Centre for Diarrheal Disease Research, Bangladesh (ICDDR, B), known as the Cholera Hospital. We are here to know about a program called Mobile Alliance for maternal action (MAMA)/ Aponjon and we knew that your daughter/daughter-in-law/wife/niece is living here in this village where this service is available and who may or may not be a user of Aponjon. We would like to talk to her about this service and would like to take your permission to include her as one of our participant for our study. Please note that we will be asking questions to her specifically about this program, what she knows about it, if she is a user of this programme or not and if not why not, what are the strengths and weaknesses of this programme and so on. The main objective of this study is to explore what people know about this program, what services they are receiving through this program and what constraints they are facing for availing the services.

Guardian gave permission Yes No

If no, please thank her/him and stop consent process there

If yes, please proceed with the informant with the following sections

**Purpose of the Research Project:**

My name is ………….............. . We work in the International Centre for Diarrheal Disease Research, Bangladesh (ICDDR, B), known as the Cholera Hospital in Dhaka where researches on different types of health issues are conducted. We are here to know about the mHealth programme on Mobile Alliance for maternal action (MAMA)/ Aponjon. We are seeking your permission to include you as a participant in this study. The objective of this study is to explore what people know about this mHealth programme, what health services they are receiving through this programme, what constraints they are facing for availing the services. Also, we would like to know how much you know about maternal and child health and how are you solving your health problems with/ without the support of various messages that you receive from different sources.

**Why you are invited to participate in the study?**

You have been asked to participate in this study because you are a mother who is using/not using the mobile based health services called Aponjon. If you agree to participate in this study we will ask you about your experiences, feelings, attitudes, and notions regarding this service. More specifically, we will seek to understand more from you regarding the maternal and child health information services through mobile phone or other sources. We would also like to know about the involvement of your husband, other family members and neighbours regarding this.

The data collection time may take from 45 minutes to 1 hour. But if you do not wish to spend all of that time at once, we can always come back at a later time. We would like to fill out a questionnaire form in order to note down the valuable information you give. The information you will provide will solely be used for this particular study and nobody besides the research investigators will be able to access it.

**Risks and benefits:**

There are no risks to you, or your baby from taking part in the study. Any information that you answer or regret to answer will have absolutely no effect on the health services you are receiving during pregnancy/delivery/post natal period or general healthcare from the nearest health facility. If you have any resentment regarding our study, then please note the contact information below and you can register your complain there.

With the help of the information you will provide by participating in this study, we will be able to understand how much beneficial the maternal and newborn health related messages that are being sent by the mHealth programme are to the people. This study will help in future in establishing mHealth policy in sending health messages to mothers of newborns. As a result maternal and newborn health will be improved.

**Privacy, anonymity and confidentiality:**

Your identity will remain confidential. Only investigators of this study will have access to study forms which will be stored in locked cabinets at our head office in Dhaka.

**Future use of information:**

Only anonymous information will be used from this interview for the objectives described to you a few moments ago. We will never use your name or your contact information in any way. Instead, we will be using an identification number so that no one can identify you.

**Right not to participate and withdraw:**

Your participation in this study is completely voluntary. You can refuse to participate in the study any time. If you don’t want to participate or if you do not wish to answer any question, you are free to refuse it.

Once you agree to participate after completely understanding the purpose of this study and your role in this project, you will be asked to sign the consent form. If you cannot sign your name, we will be requesting you to give thumbprint impression on the form. By signing the consent form you indicate that you understand the information provided here and that you give your consent to participate in this research project.

**Principle of compensation:**

Your participation is completely voluntary and you will not get any financial benefit from participating in the study. Dr. Mahbub Elahi Chowdhury is the Principle Investigator of this study. If you have any question about this study, you may contact the persons listed in the following:

**For further query please contact:**

| **Dr. Mahbub-E-Elahi Chowdhury Khan**  Scientist, Centre for Equity and health Systems,  ICDDR,B, Mohakhali, Dhaka 1212,  Phone: 9827001-10/2215  Mobile: 01713090869  E-mail: melahi@icddrb.org | M A Salam Khan  Committee coordination secretariat  ICDDR,B; Mohakhali; Dhaka 1212  Phone: 9827001-10/3206  Mobile: 01711-428989  E-mail: salamk@icddrb.org |
| --- | --- |

Do you have any questions? Yes No

Do you agree to participate in this research project? Yes No

Name of participants:_______________________________

Signature/thump impression of the participants: ___________________________________

Name of person obtaining consent: ___________________________________

Signature of Person Obtaining Consent____________________ Date______________

Signature of Person as witness of Obtaining Consent ____________Date______________

- **Screening section**

| **No** | **Questions & Filters** | **Coding** | **Responce** | **Skip** |
| --- | --- | --- | --- | --- |
| 1.1 | When did you have the delivery of your last child? (if less than 1 month write 00) | | \|__\|\|__\| | If answer is code 99 then stop the interview |
|  | - 1. ---------- months ago |  |  |  |
|  | 1. Never delivered a baby | 99 |  |  |
| 1.2 | Is your last delivered baby still alive? | | \|__\| | If answer is code 2 or 3 then stop the interview |
|  | 1. Yes | 1 |  |  |
|  | 1. No | 2 |  |  |
|  | 1. Stillborn | 3 |  |  |
| 1.3 | Do you have your own mobile phone? | | \|__\| | If answer is code 1 then skip to Q 1.6 |
|  | 1. Yes | 1 |  |  |
|  | 1. No | 2 |  |  |
| 1.4 | In case of your need, can you access any mobile phone of others? | | \|__\| | If answer is code 2 then stop the interview |
|  | 1. Yes | 1 |  |  |
|  | 1. No | 2 |  |  |
| 1.5 | Whose mobile phone do you use in case of your need? (multiple answer) Yes=1. No=2 | |  |  |
|  | 1. Husband’s mobile phone | | \|__\| |  |
|  | 1. Mother’s mobile phone | | \|__\| |  |
|  | 1. Father’s mobile phone | | \|__\| |  |
|  | 1. Mother-in-law’s mobile phone | | \|__\| |  |
|  | 1. Brother/sister’s mobile phone | | \|__\| |  |
|  | 1. Brother-in-law/sister-in-law’s mobile phone | | \|__\| |  |
|  | 1. Phone-Fax shops | | \|__\| |  |
|  | 1. Others (specify) ........................................... | | \|__\| |  |
| 1.6 | Were you ever a subscriber of Aponjon/MAMA mHealth programme? | | \|__\| | If answer is code 2 then skip to Q 1.13 |
|  | 1. Yes | 1 |  |  |
|  | 1. No | 2 |  |  |
| 1.7 | During last pregnancy period, were you a subscriber of Aponjon/MAMA mHealth programme? | | \|__\| | If answer is code 2 then skip to Q 1.10 |
|  | 1. Yes | 1 |  |  |
|  | 1. No | 2 |  |  |
| 1.8 | During the last pregnancy period, from which gestationl month did you start to receive messages from Aponjon/MAMA | | \|__\| |  |
|  | 1. 2^nd^ month of pregnancy | 1 |  |  |
|  | 1. 3^rd^ month of pregnancy | 2 |  |  |
|  | 1. 4^th^ month of pregnancy | 3 |  |  |
|  | 1. 5^th^ month of pregnancy | 4 |  |  |
|  | 1. 6^th^ month of pregnancy | 5 |  |  |
|  | 1. 7^th^ month of pregnancy | 6 |  |  |
|  | 1. 8^th^ month of pregnancy | 7 |  |  |
|  | 1. 9^th^ month of pregnancy | 8 |  |  |
| 1.9 | During the last pregnancy, how many months in total were you subscribed to Aponjon/MAMA mHealth programme? | | \|__\|\|__\| |  |
|  | - 1. ---------- months |  |  |  |
|  | - 1. I don’t remember/I don’t know | 99 |  |  |
| 1.10 | After the last delivery, were you a subscriber of Aponjon/MAMA mHealth programme during the postnatal period? | | \|__\| | If answer is code 2 then skip to Q1.13 |
|  | 1. Yes | 1 |  |  |
|  | 1. No | 2 |  |  |
| 1.11 | After the last delivery, from which postnatal month did you start to receive messages from Aponjon/MAMA | | \|__\| |  |
|  | 1. 1^st^ postnatal month | 1 |  |  |
|  | 1. 2^nd^ postnatal month | 2 |  |  |
|  | 1. 3^rd^ postnatal month | 3 |  |  |
|  | 1. 4^th^ postnatal month | 4 |  |  |
|  | 1. 5^th^ postnatal month | 5 |  |  |
|  | 1. 6^th^ postnatal month | 6 |  |  |
| 1.12 | After the last delivery, how many months in total were you subscribed to Aponjon/MAMA mHealth programme? | | \|__\| |  |
|  | 1. ---------- months |  |  |  |
|  | 1. I don’t remember/I don’t know | 9 |  |  |

**Instruction for the interviewer: circle USER and NON-USER group as per the criteria set in the table below**

| Criteria for mothers of 7-12 months babies who are USER   1. Had delivered a baby within last 7-12 months 2. Respondent has own mobile phone or has access to mobile phone of any other member of the household 3. Resident of that locality for at least 6 months 4. After the last delivery, was subscribed to Aponjon/MAMA for at least 3 months during first 6 months postnatal period.   If all 4 criteria mentioned above is fulfilled then include the mother as Aponjon USER  Criteria for mother of 7-12 months babies who are NON- USER   1. Had delivered a baby within last 7-12 months 2. Respondent has own mobile phone or has access to mobile phone of any other member of the household 3. Resident of that locality for at least 6 months 4. After the last delivery, was never a subscriber of Aponjon/MAMA during first 6 months postnatal period   If all 4 criteria mentioned above is fulfilled then include the mother as Aponjon NON USER | **Aponjon USER------1**  Aponjon NON USER**-----2** |
| --- | --- |

**SECTION 1: INDIVIDUAL CHARACTERISTICS**

1. **INFORMATION ON THE SKILLS OF OPERATING MOBILE PHONE**

| **No** | **Questions & Filters** | **Coding** | **Responce** | **Skip** |
| --- | --- | --- | --- | --- |
| 1.13 | Do you know how to make calls from mobile phone? | | \|__\| |  |
|  | 1. Yes | 1 |  |  |
|  | 1. No | 2 |  |  |
| 1.14 | Do you know how to receive calls in mobile phone? | | \|__\| |  |
|  | 1. Yes | 1 |  |  |
|  | 1. No | 2 |  |  |
| 1.15 | Do you how to write and send SMS from mobile phone | | \|__\| |  |
|  | 1. Yes | 1 |  |  |
|  | 1. No | 2 |  |  |
| 1.16 | Do you know how to open and read the received SMS in mobile phone? | | \|__\| |  |
|  | 1. Yes | 1 |  |  |
|  | 1. No | 2 |  |  |
| 1.17 | Do you have mobile network available in the area you live in? | | \|__\| | If answer is code 1 or 3 skip to Q 1.19 |
|  | 1. Yes. Always available | 1 |  |  |
|  | 1. Yes, sometimes available | 2 |  |  |
|  | 1. No, never available | 3 |  |  |
| 1.18 | What are the problems you face for often not getting mobile network? (multiple answer) Yes=1, No=2 | |  |  |
|  | 1. Cant hear clearly | | \|__\| |  |
|  | 1. Calls get disconnected frequently | | \|__\| |  |
|  | 1. Cant make calls when needed | | \|__\| |  |
|  | 1. Cant receive calls when needed | | \|__\| |  |
|  | 1. Cant send SMS when needed | | \|__\| |  |
|  | 1. Others (specify)   _____________­­­­­­­­­­­­­­­­­­­­­__________________ | | \|__\| |  |

1. **EXPOSURE TO OTHER SOURCE OF HEALTH MESSAGES**

| **No** | **Questions & Filters** | **Coding** | **Responce** | **Skip** |
| --- | --- | --- | --- | --- |
| 1.19 | Do you have any of the following mass media in your home? (Read each of them) Yes=1, No=2 | |  |  |
|  | 1. Newspaper | | \|__\| |  |
|  | 1. Magazine | | \|__\| |  |
|  | 1. Radio | | \|__\| |  |
|  | 1. Television | | \|__\| |  |
|  | 1. Internet | | \|__\| |  |
| 1.20 | From where did you receive messages on maternal and newborn healthcare during pregnancy/delivery/postnatal period? (multiple answer) Yes=1, No=2 | |  |  |
|  | 1. Govt. hospital (Medical college hospital/district hospital/MCWC/upazila health complex) | | \|__\| |  |
|  | 1. Govt. health centre (UH&FWC/union sub centre) | | \|__\| |  |
|  | 1. Community clinic | | \|__\| |  |
|  | 1. Satellite clinic | | \|__\| |  |
|  | 1. Private hospital/clinic | | \|__\| |  |
|  | 1. NGO clinic | | \|__\| |  |
|  | 1. Private chamber of medical doctors | | \|__\| |  |
|  | 1. SMS from Govt. | | \|__\| |  |
|  | 1. EPI session/campaign | | \|__\| |  |
|  | 1. mHealth programme (write the name of the programme if known ___________________________ ) | | \|__\| |  |
|  | 1. Community meeting | | \|__\| |  |
|  | 1. Mass media | | \|__\| |  |
|  | 1. Community health workers | | \|__\| |  |
|  | 1. Family members | | \|__\| |  |
|  | 1. Others (specify) _______________________________ | | \|__\| |  |

**SECTION 2: KNOWLEDGE ON ‘APONJON”**

**A. KNOWLEDGE OF BOTH USER AND NON USER ON “APONJON SERVICE”**

| **No** | **Questions and Filters** | **Coding** | **Responce** | **Skip** |
| --- | --- | --- | --- | --- |
| 2.1 | Have you heard about Aponjon/MAMA mHealth programme? | | \|__\| |  |
|  | 1. Yes | 1 |  |  |
|  | 1. No | 2 |  |  |
| **(In case of Aponjon USER if answer is code 2, then inform her about the name Aponjon/MAMA of the mHealth programme she is subscribed to and go to next question. In case of Aponjon NON-USER if answer is code 2 then skip to section 4)** | | | | |
| 2.2 | From where/whom did you hear about Aponjon/MAMA mHealth programme? (multiple answer) Yes=1, No=2 | |  |  |
|  | 1. Community health worker | | \|__\| |  |
|  | 1. Healthcare provider of Govt. health facility | | \|__\| |  |
|  | 1. Healthcare provider of private health facility | | \|__\| |  |
|  | 1. Family members | | \|__\| |  |
|  | 1. Friends/neighbours/relatives | | \|__\| |  |
|  | 1. Other Aponjon users | | \|__\| |  |
|  | 1. Television | | \|__\| |  |
|  | 1. Internet | | \|__\| |  |
|  | 1. Newspapers | | \|__\| |  |
|  | 1. Radio | | \|__\| |  |
|  | 1. Leaflet/poster | | \|__\| |  |
|  | 1. Announcements/miking | | \|__\| |  |
|  | 1. Community meeting | | \|__\| |  |
|  | 1. Others (specify) _____________________________ __________ | | \|__\| |  |
| 2.3 | What kind of health messages the Aponjon/MAMA mHealth programme provides? (multiple answers) Yes=1, No=2 | |  |  |
|  | 1. Antenatal/delivery care related information | | \|__\| |  |
|  | 1. Symptoms of pregnancy/delivery complications | | \|__\| |  |
|  | 1. Necessity of birth planning | | \|__\| |  |
|  | 1. Information on immunization during pregnancy | | \|__\| |  |
|  | 1. Postnatal care related information | | \|__\| |  |
|  | 1. Newborn care related information | | \|__\| |  |
|  | 1. Information on mental development of child | | \|__\| |  |
|  | 1. Prevention and management of diarrhoea of child | | \|__\| |  |
|  | 1. Sympotoms of newborn health complications | | \|__\| |  |
|  | 1. Source of maternal and newborn healthcare | | \|__\| |  |
|  | 1. Benefit of breast feeding | | \|__\| |  |
|  | 1. Information on maternal nutrition | | \|__\| |  |
|  | 1. Information on child nutrition | | \|__\| |  |
|  | 1. Information on immunization of child | | \|__\| |  |
|  | 1. Family planning advice | | \|__\| |  |
|  | 1. Hygeine advice | | \|__\| |  |
|  | 1. I don’t know | | \|__\| |  |
|  | 1. Others (specify) _______________________________ | | \|__\| |  |
| 2.4 | What are the ways through which Aponjon/MAMA mHealth programme delivers health messages (multiple answer) Yes=1, No=2. | |  |  |
|  | 1. SMS | | \|__\| |  |
|  | 1. IVR push | | \|__\| |  |
|  | 1. IVR pull | | \|__\| |  |
|  | 1. I don’t know | | \|__\| |  |
| 2.5 | Do you know, if one needs to pay any money to receive health messages from Aponjon/MAMA mHealth programme? | | \|__\| | If answer is code 2 or 9 skip to Q  2.7 |
|  | 1. Yes | 1 |  |  |
|  | 1. No | 2 |  |  |
|  | 1. I don’t know | 9 |  |  |
| 2.6 | What is the cost of each health message provided by Aponjon/MAMA mHealth programme | | \|__\|.\|__\|\|__\| |  |
|  | 1. ..............:.................... Taka |  |  |  |
|  | 1. I don’t know | 999 |  |  |
| 2.7 | Does Aponjon/MAMA mHealth programme provides messages at subsidy/free of cost for the poor? | | \|__\| |  |
|  | 1. Yes | 1 |  |  |
|  | 1. No | 2 |  |  |
|  | 1. I don’t know | 9 |  |  |

1. **KNOWLEDGE OF APONJON USERS ABOUT THE SERVICE**

***** For Aponjon non users skip to Section 4***

| **No** | **Questions & Filters** | **Coding** | | | **Response** | **Skip** |
| --- | --- | --- | --- | --- | --- | --- |
| 2.8 | During last postnatal period, besides family members who else encouraged you to subscribe Aponjon/MAMA mHealth programme? (multiple answer) Yes=1, No=2 | | | |  |  |
|  | 1. Community health worker | | | | \|__\| |  |
|  | 1. Other Aponjon user | | | | \|__\| |  |
|  | 1. Provider of health facility | | | | \|__\| |  |
|  | 1. No one encouraged me | | | | \|__\| |  |
|  | 1. Others (specify) _________________________________ | | | | \|__\| |  |
| 2.9 | During last postnatal period, how did you register Aponjon/MAMA mHealth programme? | | | | \|__\| | If answer is code 1 then skip to Q 2.17  If answer is code 2/3/4/5 then skip to Q 2.11 |
|  | 1. I started receving messages without registering for the programme | 1 | | |  |  |
|  | 1. Registration done by myself | 2 | | |  |  |
|  | 1. Registration done by husband | 3 | | |  |  |
|  | 1. Registration done by other family members | 4 | | |  |  |
|  | 1. Registration done by friend/neighbour | 5 | | |  |  |
|  | 1. Registration done by community health worker | 6 | | |  |  |
|  | 1. Registration done by care provider of hospital | 7 | | |  |  |
| 2.10 | During last postnatal period, did the community health worker/care provider of hospital take your permission before registering you to the Aponjon/MAMA mHealth programme? | | | | \|__\| | skip to Q 2.13 |
|  | 1. Yes | 1 | | |  |  |
|  | 1. No | 2 | | |  |  |
| 2.11 | During last postnatal period, did you face any difficulty while registering Aponjon/MAMA mHealth programme? | | | | \|__\| | If answer is code 2 then skip to Q 2.13 |
|  | 1. Yes | 1 | | |  |  |
|  | 1. No | 2 | | |  |  |
| 2.12 | During last postnatal period, why you faced difficulty while registering Aponjon/MAMA mHealth programme? (multiple answer) Yes=1, No=2 | | | |  |  |
|  | 1. Community health workers did not explain the registration process properly | | | | \|__\| |  |
|  | 1. Registration process was complicated | | | | \|__\| |  |
|  | 1. Registration instructions were too lengthy to follow | | | | \|__\| |  |
|  | 1. Other (specify) _________________________ | | | | \|__\| |  |
| 2.13 | During last postnatal period, whose mobile phone did you use to subscribe Aponjon/MAMA mHealth programme? | | | | \|__\| | If answer is code 1 then skip to Q 2.16 |
|  | 1. My own mobile phone | 1 | | |  |  |
|  | 1. Husband’s mobile phone | 2 | | |  |  |
|  | 1. Mother/father’s mobile phone | 3 | | |  |  |
|  | 1. Mother-in-law/father-in-law’s mobile phone | 4 | | |  |  |
|  | 1. Brother/sister’s mobile phone | 5 | | |  |  |
|  | 1. Brother-in-law/sister-in-law’s mobile phone | 6 | | |  |  |
|  | 1. Other (specify) _________________________ | 7 | | |  |  |
| 2.14 | During last postnatal period, why didn’t you use your own mobile phone for subscription of Aponjon/MAMA mHealth programme? (multiple answer) Yes=1, No=2 | | | |  |  |
|  | 1. I didn’t have own mobile | | | | \|__\| |  |
|  | 1. I didn’t know my mobile number to give to Community health worker | | | | \|__\| |  |
|  | 1. My mobile phone was out-of-order | | | | \|__\| |  |
|  | 1. I don’t know how to operate mobile phone | | | | \|__\| |  |
|  | 1. Other (specify) __________________________ | | | | \|__\| |  |
| 2.15 | During last postnatal period, did you face any problem for subscribing Aponjon/MAMA mHealth programme in other’s mobile phone? (multiple answer) Yes=1, No=2 | | | |  |  |
|  | 1. There was no problem | | | | \|__\| |  |
|  | 1. I didn’t have acces to that mobile phone all the time | | | | \|__\| |  |
|  | 1. Others forgot to inform me about the messages | | | | \|__\| |  |
|  | 1. Others were irritated for the cost of health messages | | | | \|__\| |  |
|  | 1. Other (specify) _______________________ | | | | \|__\| |  |
| 2.16 | During last postnatal period, who was the decision maker for your subscription of Aponjon/MAMA mHealth programme? | | | | \|__\| | Skip to Q 2.18 |
|  | 1. Myself | 1 | | |  |  |
|  | 1. Husband | 2 | | |  |  |
|  | 1. Mother | 3 | | |  |  |
|  | 1. Mother-in-law | 4 | | |  |  |
|  | 1. Father | 5 | | |  |  |
|  | 1. Father-in-law | 6 | | |  |  |
|  | 1. Other family members | 7 | | |  |  |
|  | 1. Other (specify) _______________________ | 8 | | |  |  |
| 2.17 | During last postnatal period, in whose mobile phone the messages came that you received without registering with the Aponjon/MAMA mHealth programme? | | | | \|__\| |  |
|  | 1. Self | 1 | | |  |  |
|  | 1. Husband’s | 2 | | |  |  |
|  | 1. Father/mother’s | 3 | | |  |  |
|  | 1. Father-in-law/mother-in-law’s | 4 | | |  |  |
|  | 1. Brother/sister’s | 5 | | |  |  |
|  | 1. Brother-in-law/sister-in-law’s | 6 | | |  |  |
|  | 1. Other (specify) _________________________ | 7 | | |  |  |
| 2.18 | During last postnatal period, how many messages you used to receive per week from Aponjon/MAMA mHealth programme? | | | | \|__\| |  |
|  | 1. 1-2 messages | 1 | | |  |  |
|  | 1. 3-5 messges | 2 | | |  |  |
|  | 1. 6 or more messges | 3 | | |  |  |
| 2.19 | During last postnatal period, did you regularly read/listen all the messages sent by Aponjon/MAMA mHealth programme? | | | | \|__\| |  |
|  | 1. Read/listened all messages regularly | 1 | | |  |  |
|  | 1. Read/listened few messages | 2 | | |  |  |
|  | 1. Did not read/listen most of the messages | 3 | | |  |  |
| 2.20 | Do you know upto how many months Aponjon/MAMA mHealth programme sends messages during pregnancy period? (if answer is upto delivery of the baby write code 09) | | | | \|__\|\|__\| |  |
|  | 1. Upto ……………….. months of pregnancy |  | | |  |  |
|  | 1. I don’t know | 99 | | |  |  |
| 2.21 | Do you know upto how many months Aponjon/MAMA mHealth programme sends messages during postnatal period? | | | | \|__\|\|__\| |  |
|  | 1. Upto ……………….. months postnatally |  | | |  |  |
|  | 1. I don’t know | 99 | | |  |  |
| 2.22 | During last postnatal period, what are the benefits you had due to subscribing Aponjon/MAMA mHealth programme? (multiple answer) Yes=1, No=2 | | | |  |  |
|  | 1. I received necessary information on my own healthcare | | | | \|__\| |  |
|  | 1. I received necessary information on my child’s healthcare | | | | \|__\| |  |
|  | 1. I was able to receive information easily at my own home | | | | \|__\| |  |
|  | 1. I was able to learn new information about healthcare | | | | \|__\| |  |
|  | 1. Other (specify) _____________________________ | | | | \|__\| |  |
| 2.23 | During last postnatal period, in which method you received messages from Aponjon/MAMA mHealth programme | | | | \|__\| | If answer is code 2 skip to 2.28  And  if answer is code 3 skip to 2.35 |
|  | 1. SMS | 1 | | |  |  |
|  | 1. IVR push | 2 | | |  |  |
|  | 1. IVR pull | 3 | | |  |  |
| **FOR SMS CLIENTS (**** ***Skip this section for IVR clients to 2.28)*** | | | | | | |
| 2.24 | During last postnatal period, why did you choose to receive messages through SMS from Aponjon/MAMA mHealth programme? (multiple answer) Yes=1, No=2 | | | |  |  |
|  | 1. Health worker specified this method for me | | | | \|__\| |  |
|  | 1. I knew about this method only | | | | \|__\| |  |
|  | 1. I can read and understand SMS | | | | \|__\| |  |
|  | 1. I can read SMS again and again if needed | | | | \|__\| |  |
|  | 1. I can read SMS when I am free | | | | \|__\| |  |
|  | 1. I can receive SMS even if there is network problem | | | | \|__\| |  |
|  | 1. I can ask someone to explain the message if I am unable to understand | | | | \|__\| |  |
|  | 1. Other (specify)   _______________________ | | | | \|__\| |  |
| 2.25 | During last postnatal period, did you yourself read the health messages sent by SMS or someone else read it to you? | | | | \|__\| |  |
|  | 1. I read it myself | 1 | | |  |  |
|  | 1. Someone else read it to me | 2 | | |  |  |
| 2.26 | During last postnatal period, did you face any problem in understanding the health messages sent by SMS? | | | | \|__\| | If answer is code 2 skip to section 3 |
|  | 1. Yes | 1 | | |  |  |
|  | 1. No | 2 | | |  |  |
| 2.27 | During last postnatal period, what kind of problem you faced in understanding the messages sent by SMS? (multiple answer) Yes=1, No=2 | | | |  | ‡mKkb 3 G hvb |
|  | 1. It was difficult to understand Bangla messages in English script/Banglish writing | | | | \|__\| |  |
|  | 1. Messages were not understandable if not read repeatedly | | | | \|__\| |  |
|  | 1. It was not clear that the SMS was sent by Aponjon/MAMA mHealth programme | | | | \|__\| |  |
|  | 1. Other (specify)   ___________________________ | | | | \|__\| |  |
| **FOR PUSH MODE IVR CLIENTS (receive calls from Aponjon)**  *****For Pull Mode clients please skip to 2 .35*** | | | | | | |
| 2.28 | During last postnatal period, why did you choose to receive messages through IVR push method? (multiple answer) Yes=1, No=2 | | |  | |  |
|  | 1. Health worker specified this method for me | | | \|__\| | |  |
|  | 1. I was aware of this method only | | | \|__\| | |  |
|  | 1. I could listen the to the voice of the lady doctor directly | | | \|__\| | |  |
|  | 1. I don’t know how to open and read SMS in mobile phone | | | \|__\| | |  |
|  | 1. I know how to receive calls only in mobile phone | | | \|__\| | |  |
|  | 1. Other (specify)   _____________________________ | | | \|__\| | |  |
| 2.29 | During last postnatal period, did you have any option to choose your preferred time to receive messages during registration with Aponjon/MAMA mHealth programme? | | | \|__\| | | If answer is code 2 or 9 then skip to Q 2.32 |
|  | 1. Yes | | 1 |  |  |  |
|  | 1. No | | 2 |  |  |  |
|  | 1. I don’t know | | 9 |  |  |  |
| 2.30 | During last postnatal period, what time did you choose to receive messages from Aponjon/MAMA mHealth programme? | | | \|__\| | |  |
|  | 1. Morning | | 1 |  |  |  |
|  | 1. Afternoon | | 2 |  |  |  |
|  | 1. Evening | | 3 |  |  |  |
|  | 1. Night | | 4 |  |  |  |
|  | 1. Any time of the day | | 5 |  |  |  |
| 2.31 | During last postnatal period, did you receive messages according to your preferred time? | | | \|__\| | |  |
|  | 1. Yes | | 1 |  |  |  |
|  | 1. No | | 2 |  |  |  |
| 2.32 | During last postnatal period, did you usually used to miss the call from Aponjon/MAMA mHealth programme? | | | \|__\| | | If answer is code 2 then skip to section 3 |
|  | 1. Yes | | 1 |  |  |  |
|  | 1. No | | 2 |  |  |  |
| 2.33 | During last postnatal period, what were reasons that you missed calls from Aponjon/MAMA mHealth programme? (multiple answer) Yes=1, No=2 | | |  | |  |
|  | 1. Not having balance in the mobile | | | \|__\| | |  |
|  | 1. Being busy in household works | | | \|__\| | |  |
|  | 1. Someone else had the mobile phone | | | \|__\| | |  |
|  | 1. Messages did not come at my preferred time | | | \|__\| | |  |
|  | 1. Someone else may have received the call | | | \|__\| | |  |
|  | 1. It costs money to receive calls from Amonjon | | | \|__\| | |  |
|  | 1. Other (specify)   ______________________________ | | | \|__\| | |  |
| 2.34 | During last postnatal period, what did you do if you ever missed call from Aponjon/MAMA mHealth programme? | | | \|__\| | | Skip to section 3 |
|  | 1. I didn’t do anything | | |  |  |  |
|  | 1. I waited for the next call to receive the message | | |  |  |  |
|  | 1. I called Aponjon to listen to the message | | |  |  |  |
|  | 1. Other (specify)   ________________________________ | | |  |  |  |
| **PULL MODE IVR (Call Aponjon for listening to messages)** | | | | | | |
| 2.35 | During last postnatal period, why did you choose to receive messages through IVR pull method?  (multiple answer) Yes=1, No=2 | | | |  |  |
|  | 1. Health worker specified this method for me | | | | \|__\| |  |
|  | 1. I was aware of this method only | | | | \|__\| |  |
|  | 1. It is easier to receive health messages in this method | | | | \|__\| |  |
|  | 1. I don’t know how to open and read SMS in mobile phone | | | | \|__\| |  |
|  | 1. I can make call in my preferred time | | | | \|__\| |  |
|  | 1. I could listen the to the voice of the lady doctor directly | | | | \|__\| |  |
|  | 1. Other (specify)   _________________________________ | | | | \|__\| |  |

**SECTION 3: QUESTIONS EXPLORED WITH THE CLIENT ON HER GATEKEEPER (HUSBAND/GUARDIAN)**

| **No** | **Questions and Filters** | **Coding** | **Response** | **Skip** |
| --- | --- | --- | --- | --- |
| 3.1 | During last postnatal period, was your husband/guardian also subscribed Aponjon/MAMA mHealth programme as gatekeeper? | | \|__\| | If answer is code 2 or 9 then skip to Section 4 |
|  | 1. Yes | 1 |  |  |
|  | 1. No | 2 |  |  |
|  | 1. I don’t know | 9 |  |  |
| 3.2 | During last postnatal period, did the health worker took your/his/her permission before registering your husband/guardian as gatekeep in Aponjon/MAMA mHealth programme? | | \|__\| |  |
|  | 1. Yes | 1 |  |  |
|  | 1. No | 2 |  |  |
|  | 1. We registered ourselves so didn’t require their permission | 3 |  |  |
|  | 1. I don’t remember/I don’t know | 9 |  |  |
| 3.3 | During last postnatal period, did you get any benefit by including your husband/guardian as gatekeeper in Aponjon/MAMA mHealth programme? | | \|__\| | If answer is code 2 then skip to 3.5 |
|  | 1. Yes | 1 |  |  |
|  | 1. No | 2 |  |  |
| 3.4 | During last postnatal period, what were the benefits you got by including your husband/guardian as gatekeeper in Aponjon/MAMA mHealth programme? (multiple answer) Yes=1, No=2 | |  | Skip to section 4 |
|  | 1. He/she helped me in understanding the health messages | | \|__\| |  |
|  | 1. He/she helped me in following the instructions of the health messages | | \|__\| |  |
|  | 1. He/she used to be aware about the need of healthcare for me | | \|__\| |  |
|  | 1. As decision maker he/she needed to know about the healthcare during postnatal period | | \|__\| |  |
|  | 1. Other (specify)   _________________________________ | | \|__\| |  |
| 3.5 | During last postnatal period, what were the problems you faced for including your husband/guardian as gatekeeper in Aponjon/MAMA mHealth programme? (multiple answer) Yes=1, No=2 | |  |  |
|  | 1. Expenses were double for the cost of messages of both me and my gatekeeper | | \|__\| |  |
|  | 1. Husband/guardian was not interested to discuss the health messages of Aponjon | | \|__\| |  |
|  | 1. Husband/guardian did not take any initiative to help follow the advices of Aponjon | | \|__\| |  |
|  | 1. There was no need to get same messages for me and my gatekeeper | | \|__\| |  |
|  | 1. Other (specify)   _______________________ | | \|__\| |  |

**SECTION 4: KNOWLEDGE AND PRACTICES ON MATERNAL AND NEWBORN HEALTH CARE AND CARE SEEKING BEHAVIOUR**

| No | Questions and Filters | | | | | Coding | | | | | Responce | | Skip |
| --- | --- | --- | --- | --- | --- | --- | --- | --- | --- | --- | --- | --- | --- |
| 4.1 | What are the places where delivery is conducted by skilled/trained personnel? (multiple answer) Yes=1, No=2 | | | | | | | | | |  | |  |
|  | 1. Govt. hospital (Medical college hospital/district hospital/MCWC/upazila health complex) | | | | | | | | | | \|__\| | |  |
|  | 1. Govt. health centre (UH&FWC/union sub centre) | | | | | | | | | | \|__\| | |  |
|  | 1. Community clinic | | | | | | | | | | \|__\| | |  |
|  | 1. Private hospital/clinic | | | | | | | | | | \|__\| | |  |
|  | 1. NGO clinic | | | | | | | | | |  | |  |
|  | 1. BRAC delivery centre | | | | | | | | | |  | |  |
|  | 1. Private chamber of medical doctors | | | | | | | | | | \|__\| | |  |
|  | 1. CSBA/FWV/nurse/paramedic’s home | | | | | | | | | | \|__\| | |  |
|  | 1. Own home by skilled/trained provider | | | | | | | | | | \|__\| | |  |
|  | 1. Own home by NGO worker | | | | | | | | | |  | |  |
|  | 1. Own home by unskilled provider (TTBA/TBA/relative/family member/neighbours) | | | | | | | | | | \|__\| | |  |
|  | 1. TTBA/TBA’s house | | | | | | | | | | \|__\| | |  |
|  | 1. Village doctor’s house | | | | | | | | | | \|__\| | |  |
|  | 1. Homeo doctor’s house | | | | | | | | | | \|__\| | |  |
|  | 1. Traditional healer’s house | | | | | | | | | | \|__\| | |  |
|  | 1. I don’t know | | | | | | | | | | \|__\| | |  |
|  | 1. Other (specify)   ............................................................. | | | | | | | | | | \|__\| | |  |
| 4.2 | How far is the nearest facility from your home where normal delivery care is available? (if less than 1 hour write code 00) | | | | | | | | | | \|__\|\|__\| | |  |
|  | 1. ----------- hour | | | | |  | | | | |  |  |  |
|  | 1. I don’t know | | | | | 99 | | | | |  |  |  |
| 4.3 | How far is the nearest facility from your home where C-section delivery care is available? (if less than 1 hour write code 00) | | | | | | | | | | \|__\|\|__\| | |  |
|  | 1. ----------- hour | | | | |  | | | | |  |  |  |
|  | 1. I don’t know | | | | | 99 | | | | |  |  |  |
| 4.4 | Where was your last child delivered? | | | | |  | | | | | \|__\|\|__\| | | If answer is any of the code 6 to 16 then skip to Q 4.6 |
|  | 1. Govt. hospital (Medical college hospital/district hospital/MCWC/upazila health complex) | | | | | 01 | | | | |  |  |  |
|  | 1. Govt. health centre (UH&FWC/union sub centre) | | | | | 02 | | | | |  |  |  |
|  | 1. Community clinic | | | | | 03 | | | | |  |  |  |
|  | 1. Private hospital/clinic | | | | | 04 | | | | |  |  |  |
|  | 1. NGO clinic | | | | | 05 | | | | |  |  |  |
|  | 1. BRAC delivery centre | | | | | 06 | | | | |  |  |  |
|  | 1. Private chamber of medical doctors | | | | | 07 | | | | |  |  |  |
|  | 1. CSBA/FWV/nurse/paramedic’s home | | | | | 08 | | | | |  |  |  |
|  | 1. Own home by skilled/trained provider | | | | | 09 | | | | |  |  |  |
|  | 1. Own home by NGO worker | | | | | 10 | | | | |  |  |  |
|  | 1. Own home by unskilled provider (TTBA/TBA/relative/family member/neighbours) | | | | | 11 | | | | |  |  |  |
|  | 1. TTBA/TBA’s house | | | | | 12 | | | | |  |  |  |
|  | 1. Village doctor’s house | | | | | 13 | | | | |  |  |  |
|  | 1. Homeo doctor’s house | | | | | 14 | | | | |  |  |  |
|  | 1. Traditional healer’s house | | | | | 15 | | | | |  |  |  |
|  | 1. Other (specify) ............................................................. | | | | | 16 | | | | |  |  |  |
| 4.5 | Why did you go to health facility for delivery of your child? (multiple answer) Yes=1, No=2 | | | | | | | | | |  | | Skip to Q 4.7 |
|  | 1. To have safe delivery | | | | | | | | | | \|__\| | |  |
|  | 1. To give birth to a healthy baby | | | | | | | | | | \|__\| | |  |
|  | 1. Immidiate care will be available if any complication arise | | | | | | | | | | \|__\| | |  |
|  | 1. I had history previous C-section | | | | | | | | | | \|__\| | |  |
|  | 1. There are good doctor and nurses in hospital | | | | | | | | | | \|__\| | |  |
|  | 1. I don’t know | | | | | | | | | | \|__\| | |  |
|  | 1. Other (specify)   ------------------------------------------------------------------------- | | | | | | | | | | \|__\| | |  |
| 4.6 | Why didn’t you go to hospital for your delivery? (multiple answer) Yes=1, No=2 | | | | | | | | | |  | |  |
|  | 1. I couldn’t afford the cost of hospital expenses | | | | | | | | | | \|__\| | |  |
|  | 1. Doctor is not available all the time in hospital | | | | | | | | | | \|__\| | |  |
|  | 1. Hospital was very far from my home | | | | | | | | | | \|__\| | |  |
|  | 1. There was no one to take care of my other children | | | | | | | | | | \|__\| | |  |
|  | 1. Husband/other family members didn’t give permission | | | | | | | | | | \|__\| | |  |
|  | 1. Hospital authority does not allow family members to stay beside me in hospital | | | | | | | | | | \|__\| | |  |
|  | 1. It is uncomfortable to take service from male healthcare provider in the hospital | | | | | | | | | | \|__\| | |  |
|  | 1. Fear of surgery | | | | | | | | | | \|__\| | |  |
|  | 1. There was no one to take me to hospital | | | | | | | | | | \|__\| | |  |
|  | 1. There is possibility of wrong treatment in hospital | | | | | | | | | | \|__\| | |  |
|  | 1. The local TBA was more reliable than hospital | | | | | | | | | | \|__\| | |  |
|  | 1. I had no complication/I didn’t think it was necessary to deliver baby at hospital | | | | | | | | | | \|__\| | |  |
|  | 1. Others (specify) ...................................................... | | | | | | | | | | \|__\| | |  |
| 4.7 | What are the complications/danger signs during postnatal period? (multiple answer) Yes=1, No=2 | | | | | | | | | |  | |  |
|  | 1. Severe Hemorrhage | | | | | | | | | | \|__\| | |  |
|  | 1. Severe headache | | | | | | | | | | \|__\| | |  |
|  | 1. Blurring of vision | | | | | | | | | | \|__\| | |  |
|  | 1. Convulsion | | | | | | | | | | \|__\| | |  |
|  | 1. High fever | | | | | | | | | | \|__\| | |  |
|  | 1. Anaemia | | | | | | | | | | \|__\| | |  |
|  | 1. Back pain | | | | | | | | | | \|__\| | |  |
|  | 1. Severe lower abdominal pain | | | | | | | | | | \|__\| | |  |
|  | 1. Foul smelling vaginal discharge | | | | | | | | | | \|__\| | |  |
|  | 1. I don’t know | | | | | | | | | | \|__\| | |  |
|  | 1. Others (specify) ...................................................... | | | | | | | | | | \|__\| | |  |
| 4.8 | What are the sources where care is available if any of the postnatal complications/danger sign arise? (multiple answer) Yes=1, No=2 | | | | | | | | | |  | |  |
|  | 1. Govt. hospital (Medical college hospital/district hospital/MCWC/upazila health complex) | | | | | | | | | | \|__\| | |  |
|  | 1. Govt. health centre (UH&FWC/union sub centre) | | | | | | | | | | \|__\| | |  |
|  | 1. Community clinic | | | | | | | | | | \|__\| | |  |
|  | 1. Sattelite clinic | | | | | | | | | | \|__\| | |  |
|  | 1. Private hospital/clinic | | | | | | | | | | \|__\| | |  |
|  | 1. NGO clinic | | | | | | | | | | \|__\| | |  |
|  | 1. Private chamber of medical doctors | | | | | | | | | | \|__\| | |  |
|  | 1. CSBA/FWV/nurse/paramedic’s home | | | | | | | | | | \|__\| | |  |
|  | 1. Own home by skilled/trained provider | | | | | | | | | | \|__\| | |  |
|  | 1. Own home by NGO worker | | | | | | | | | | \|__\| | |  |
|  | 1. TTBA/TBA’s house | | | | | | | | | | \|__\| | |  |
|  | 1. Village doctor’s house | | | | | | | | | | \|__\| | |  |
|  | 1. Homeo doctor’s house | | | | | | | | | | \|__\| | |  |
|  | 1. Traditional healer’s house | | | | | | | | | | \|__\| | |  |
|  | 1. Pharmacy/medicine shop | | | | | | | | | | \|__\| | |  |
|  | 1. I don’t know | | | | | | | | | | \|__\| | |  |
|  | 1. Others (specify) ....................................................... | | | | | | | | | | \|__\| | |  |
| 4.9 | During last postnatal period, did you have any complications/danger sign? | | | | | | | | | | \|__\| | | If answer is code 2, skip to Q 4.14 |
|  | 1. Yes | | | | | | | 1 | | |  |  |  |
|  | 1. No | | | | | | | 2 | | |  |  |  |
| 4.10 | During last postnatal period, what are the complications/danger signs you had? (multiple answer) Yes=1, No=2 | | | | | | | | | |  | |  |
|  | 1. Severe Hemorrhage | | | | | | | | | | \|__\| | |  |
|  | 1. Severe headache | | | | | | | | | | \|__\| | |  |
|  | 1. Blurring of vision | | | | | | | | | | \|__\| | |  |
|  | 1. Convulsion | | | | | | | | | | \|__\| | |  |
|  | 1. High fever | | | | | | | | | | \|__\| | |  |
|  | 1. Anaemia | | | | | | | | | | \|__\| | |  |
|  | 1. Back pain | | | | | | | | | | \|__\| | |  |
|  | 1. Severe lower abdominal pain | | | | | | | | | | \|__\| | |  |
|  | 1. Foul smelling vaginal discharge | | | | | | | | | | \|__\| | |  |
|  | 1. Others (specify) ...................................................... | | | | | | | | | | \|__\| | |  |
| 4.11 | During last postnatal period, did you seek care for the complications/danger signs you had? | | | | | | | | | | \|__\| | | DËi 2 †KvW n‡j 4.13 cÖ‡kœ hvb |
|  | 1. Yes | | | | | | | | 1 | |  |  |  |
|  | 1. No | | | | | | | | 2 | |  |  |  |
| 4.12 | During last postnatal period, from where did you seek care for complications/danger signs that you had?  (multiple answer) Yes=1, No=2 | | | | | | | | | |  | | Skip to Q 4.14 |
|  | 1. Govt. hospital (Medical college hospital/district hospital/MCWC/upazila health complex) | | | | | | | | | | \|__\| | |  |
|  | 1. Govt. health centre (UH&FWC/union sub centre) | | | | | | | | | | \|__\| | |  |
|  | 1. Community clinic | | | | | | | | | | \|__\| | |  |
|  | 1. Sattelite clinic | | | | | | | | | | \|__\| | |  |
|  | 1. Private hospital/clinic | | | | | | | | | | \|__\| | |  |
|  | 1. NGO clinic | | | | | | | | | | \|__\| | |  |
|  | 1. Private chamber of medical doctors | | | | | | | | | | \|__\| | |  |
|  | 1. CSBA/FWV/nurse/paramedic’s home | | | | | | | | | | \|__\| | |  |
|  | 1. Own home by skilled/trained provider | | | | | | | | | | \|__\| | |  |
|  | 1. Own home by NGO worker | | | | | | | | | | \|__\| | |  |
|  | 1. TTBA/TBA’s house | | | | | | | | | | \|__\| | |  |
|  | 1. Village doctor’s house | | | | | | | | | | \|__\| | |  |
|  | 1. Homeo doctor’s house | | | | | | | | | | \|__\| | |  |
|  | 1. Traditional healer’s house | | | | | | | | | | \|__\| | |  |
|  | 1. Pharmacy/medicine shop | | | | | | | | | | \|__\| | |  |
|  | 1. Others (specify) ...................................................... | | | | | | | | | | \|__\| | |  |
| 4.13 | Why didn’t you seek care for the postnatal complications/danger signs you had?  (multiple answer) Yes=1, No=2 | | | | | | | | | |  | |  |
|  | 1. I didn’t know from where to seek care for postnatal complictaions | | | | | | | | | | \|__\| | |  |
|  | 1. Hopital was too far from home | | | | | | | | | | \|__\| | |  |
|  | 1. Doctor is not available all the time in hospital | | | | | | | | | | \|__\| | |  |
|  | 1. There is possibility of wrong treatment in hospital | | | | | | | | | | \|__\| | |  |
|  | 1. I couldn’t afford the cost of hospital expenses | | | | | | | | | | \|__\| | |  |
|  | 1. There was lack of support from family/husband | | | | | | | | | | \|__\| | |  |
|  | 1. There was no one to take care of my other children | | | | | | | | | | \|__\| | |  |
|  | 1. I didn’t think it was necessary | | | | | | | | | | \|__\| | |  |
|  | 1. Others (specify) ...................................................... | | | | | | | | | | \|__\| | |  |
| 4.14 | Do you think, postnatal care is required to be taken by new mothers after delivery? | | | | | | | | | | \|__\| | | If answer is code 2 or 9 skip to  Q 4.17 |
|  | 1. Yes | | | | | 1 | | | | |  |  |  |
|  | 1. No | | | | | 2 | | | | |  |  |  |
|  | 1. I don’t know | | | | | 9 | | | | |  |  |  |
| 4.15 | How many times Postnatal care (PNC) visits are required after delivery? | | | | | | | | | | \|__\|\|__\| | |  |
|  | 1. …………………………….. (number) | | | | |  | | | | |  |  |  |
|  | 1. I don’t know | | | | | 99 | | | | |  |  |  |
| 4.16 | What are the durations after delivery the PNC visits are recommended to be taken? (Multiple answer)  I don’t know=99   \|  \| Duration after birth in days/ code 99 if does not know \| \| --- \| --- \| \| 1. 1^st^ Visit \| ............................ \| \| 1. 2^nd^ Visit \| ............................ \| \| 1. 3^rd^ Visit \| ............................ \| \| 1. 4^th^ Visit \| ............................ \| | | | | | | | | | |  | |  |
|  |  |  |  |  |  |  |  |  |  |  | \|__\|\|__\| | |  |
|  |  |  |  |  |  |  |  |  |  |  | \|__\|\|__\| | |  |
|  |  |  |  |  |  |  |  |  |  |  | \|__\|\|__\| | |  |
|  |  |  |  |  |  |  |  |  |  |  | \|__\|\|__\| | |  |
| 4.17 | Do you know, where postnatal care service is available?  (multiple answer) Yes=1, No=2 | | | | | | | | | |  | |  |
|  | 1. Govt. hospital (Medical college hospital/district hospital/MCWC/upazila health complex) | | | | | | | | | | \|__\| | |  |
|  | 1. Govt. health centre (UH&FWC/union sub centre) | | | | | | | | | | \|__\| | |  |
|  | 1. Community clinic | | | | | | | | | | \|__\| | |  |
|  | 1. Sattelite clinic | | | | | | | | | | \|__\| | |  |
|  | 1. Private hospital/clinic | | | | | | | | | | \|__\| | |  |
|  | 1. NGO clinic | | | | | | | | | | \|__\| | |  |
|  | 1. Private chamber of medical doctors | | | | | | | | | | \|__\| | |  |
|  | 1. CSBA/FWV/nurse/paramedic’s home | | | | | | | | | | \|__\| | |  |
|  | 1. Own home by skilled/trained provider | | | | | | | | | | \|__\| | |  |
|  | 1. Own home by NGO worker | | | | | | | | | | \|__\| | |  |
|  | 1. TTBA/TBA’s house | | | | | | | | | | \|__\| | |  |
|  | 1. Village doctor’s house | | | | | | | | | | \|__\| | |  |
|  | 1. Homeo doctor’s house | | | | | | | | | | \|__\| | |  |
|  | 1. Traditional healer’s house | | | | | | | | | | \|__\| | |  |
|  | 1. I don’t know | | | | | | | | | | \|__\| | |  |
|  | 1. Others (specify) ....................................................... | | | | | | | | | | \|__\| | |  |
| 4.18 | Did you go for post natal care (PNC) visit? | | | | | | | | | | \|__\| | | If answer is code 2 skip to Q  4.22 |
|  | 1. Yes | | | | | 1 | | | | |  |  |  |
|  | 1. No | | | | | 2 | | | | |  |  |  |
| 4.19 | How many times you took postnatal care (PNC)? | | | | | | | | | | \|__\|\|__\| | |  |
|  | 1. …………………………….. (number) | | | | |  | | | | |  |  |  |
|  | 1. I don’t remember | | | | | 99 | | | | |  |  |  |
| 4.20 | What are the duration of PNC visits you took after delivery? (Multiple answer)  I don’t know=99   \|  \| Duration after birth in days/ code 99 if does not know \| \| --- \| --- \| \| 1. 1^st^ Visit \| ............................ \| \| 1. 2^nd^ Visit \| ............................ \| \| 1. 3^rd^ Visit \| ............................ \| \| 1. 4^th^ Visit \| ............................ \| | | | | | | | | | |  | |  |
|  |  |  |  |  |  |  |  |  |  |  | \|__\|\|__\| | |  |
|  |  |  |  |  |  |  |  |  |  |  | \|__\|\|__\| | |  |
|  |  |  |  |  |  |  |  |  |  |  | \|__\|\|__\| | |  |
|  |  |  |  |  |  |  |  |  |  |  | \|__\|\|__\| | |  |
| 4.21 | From where did you take postnatal care after delivery?  (multiple answer) Yes=1, No=2 | | | | | | | | | |  | | Skip to 4.23 |
|  | 1. Govt. hospital (Medical college hospital/district hospital/MCWC/upazila health complex) | | | | | | | | | | \|__\| | |  |
|  | 1. Govt. health centre (UH&FWC/union sub centre) | | | | | | | | | | \|__\| | |  |
|  | 1. Community clinic | | | | | | | | | | \|__\| | |  |
|  | 1. Sattelite clinic | | | | | | | | | | \|__\| | |  |
|  | 1. Private hospital/clinic | | | | | | | | | | \|__\| | |  |
|  | 1. NGO clinic | | | | | | | | | | \|__\| | |  |
|  | 1. Private chamber of medical doctors | | | | | | | | | | \|__\| | |  |
|  | 1. CSBA/FWV/nurse/paramedic’s home | | | | | | | | | | \|__\| | |  |
|  | 1. Own home by skilled/trained provider | | | | | | | | | | \|__\| | |  |
|  | 1. Own home by NGO worker | | | | | | | | | | \|__\| | |  |
|  | 1. TTBA/TBA’s house | | | | | | | | | | \|__\| | |  |
|  | 1. Village doctor’s house | | | | | | | | | | \|__\| | |  |
|  | 1. Homeo doctor’s house | | | | | | | | | | \|__\| | |  |
|  | 1. Traditional healer’s house | | | | | | | | | | \|__\| | |  |
|  | 1. Others (specify) ....................................................... | | | | | | | | | | \|__\| | |  |
| 4.22 | Why didn’t you go for post natal care visit? (multiple answer) Yes=1, No=2 | | | | | | | | | |  | |  |
|  | 1. I didn’t know from where to take PNC | | | | | | | | | | \|__\| | |  |
|  | 1. I didn’t know PNC was needed to be taken | | | | | | | | | | \|__\| | |  |
|  | 1. Due to lack of money | | | | | | | | | | \|__\| | |  |
|  | 1. Hospital was too far from home | | | | | | | | | | \|__\| | |  |
|  | 1. Doctor is not available all the time in hospital | | | | | | | | | | \|__\| | |  |
|  | 1. There was no one to take me to hospital | | | | | | | | | | \|__\| | |  |
|  | 1. Husband/other family members didn’t give permission | | | | | | | | | | \|__\| | |  |
|  | 1. There was no one to take care of my other children | | | | | | | | | | \|__\| | |  |
|  | 1. I thought it was not good to move/walk after delivery | | | | | | | | | | \|__\| | |  |
|  | 1. It was not necessary/me and my baby had no problem health wise | | | | | | | | | | \|__\| | |  |
|  | 1. Others (specify) ....................................................... | | | | | | | | | | \|__\| | |  |
| 4.23 | How to take care of umbilical core of newborn? (multiple answer) Yes=1, No=2 | | | | | | | | | |  | |  |
|  | 1. Keep it dry and don’t put anything | | | | | | | | | | \|__\| | |  |
|  | 1. Clean it with warm water | | | | | | | | | | \|__\| | |  |
|  | 1. Put oil on the cord | | | | | | | | | | \|__\| | |  |
|  | 1. Put spirit on the cord | | | | | | | | | | \|__\| | |  |
|  | 1. Put antiseptic cream on the cord | | | | | | | | | | \|__\| | |  |
|  | 1. Give warm packs | | | | | | | | | | \|__\| | |  |
|  | 1. Put boric powder on the cord | | | | | | | | | | \|__\| | |  |
|  | 1. Wash hands with soap before touching the cord | | | | | | | | | | \|__\| | |  |
|  | 1. Put Vermilion on the cord | | | | | | | | | | \|__\| | |  |
|  | 1. Put ink of mud lamp on the cord | | | | | | | | | | \|__\| | |  |
|  | 1. I don’t know | | | | | | | | | | \|__\| | |  |
|  | 1. Others (specify) ....................................................... | | | | | | | | | | \|__\| | |  |
| 4.24 | How many days after birth of the newborn the cord falls off? | | | | | | | | | | \|__\|\|__\| | |  |
|  | 1. ………….............. days after birth | | | | |  | | | | |  |  |  |
|  | 1. I don’t know | | | | | 99 | | | | |  |  |  |
| 4.25 | What are the signs and symptoms of an unhealthy/infectious umbilical cord? (multiple answer) Yes=1, No=2 | | | | | | | | | |  | |  |
|  | 1. red and swollen | | | | | | | | | | \|__\| | |  |
|  | 1. Foul smell | | | | | | | | | | \|__\| | |  |
|  | 1. Wet and wound | | | | | | | | | | \|__\| | |  |
|  | 1. Discharge blood and pus | | | | | | | | | | \|__\| | |  |
|  | 1. I don’t know | | | | | | | | | | \|__\| | |  |
|  | 1. Others (specify) ....................................................... | | | | | | | | | | \|__\| | |  |
| 4.26 | How did you take care of the umbilical cord of your newborn?? (multiple answer) Yes=1, No=2 | | | | | | | | | |  | |  |
|  | 1. Keep it dry and don’t put anything | | | | | | | | | | \|__\| | |  |
|  | 1. Clean it with warm water | | | | | | | | | | \|__\| | |  |
|  | 1. Put oil on the cord | | | | | | | | | | \|__\| | |  |
|  | 1. Put spirit on the cord | | | | | | | | | | \|__\| | |  |
|  | 1. Put antiseptic cream on the cord | | | | | | | | | | \|__\| | |  |
|  | 1. Give warm packs | | | | | | | | | | \|__\| | |  |
|  | 1. Put boric powder on the cord | | | | | | | | | | \|__\| | |  |
|  | 1. Wash hands with soap before touching the cord | | | | | | | | | | \|__\| | |  |
|  | 1. Put Vermilion on the cord | | | | | | | | | | \|__\| | |  |
|  | 1. Put ink of mud lamp on the cord | | | | | | | | | | \|__\| | |  |
|  | 1. I don’t remember | | | | | | | | | | \|__\| | |  |
|  | 1. Others (specify) ....................................................... | | | | | | | | | | \|__\| | |  |
| 4.27 | When should a newborn have bath for the first time after birth? (For those who say the day of birth/soon after birth write ‘00’ days) | | | | | | | | | | \|__\|\|__\| | |  |
|  | 1. .............. days after birth | | | | |  | | | | |  |  |  |
|  | 1. I don’t know | | | | | 99 | | | | |  |  |  |
| 4.28 | When did you bathe your newborn baby for the first time after birth?  (For those who say the day of birth/soon after birth write ‘00’ days) | | | | | | | | | | \|__\|\|__\| | |  |
|  | 1. ........... days after birth | | | | |  | | | | |  |  |  |
|  | 1. I don’t know | | | | | 99 | | | | |  |  |  |
| 4.29 | What should be given to a newborn as the first food just after birth? | | | | | | | | | | \|__\| | |  |
|  | 1. Colostrums | | | | | 1 | | | | |  |  |  |
|  | 1. breast milk without feeding the colostrums | | | | | 2 | | | | |  |  |  |
|  | 1. Bottle milk/Formula | | | | | 3 | | | | |  |  |  |
|  | 1. Sugar water | | | | | 4 | | | | |  |  |  |
|  | 1. Honey/fruit juice | | | | | 5 | | | | |  |  |  |
|  | 1. Others (specify) ....................................................... | | | | | 6 | | | | |  |  |  |
| 4.30 | What are the benefits of feeding colostrums? (multiple answer) Yes=1, No=2 | | | | | | | | | |  | |  |
|  | 1. Protect baby from diseases by improving resistence | | | | | | | | | | \|__\| | |  |
|  | 1. Gives baby energy and strength | | | | | | | | | | \|__\| | |  |
|  | 1. Beneficial for baby’s healthy growth | | | | | | | | | | \|__\| | |  |
|  | 1. Baby becomes intelligent/brilliant | | | | | | | | | | \|__\| | |  |
|  | 1. Baby’s health becomes good | | | | | | | | | | \|__\| | |  |
|  | 1. I don’t know | | | | | | | | | | \|__\| | |  |
|  | 1. Others (specify) ....................................................... | | | | | | | | | | \|__\| | |  |
| 4.31 | Upto what age in months a child should be exclusively breast fed? | | | | | | | | | | \|__\|\|__\| | |  |
|  | 1. Upto ........................ months age | | | | |  | | | | |  |  |  |
|  | 1. I don’t know | | | | | 99 | | | | |  |  |  |
| 4.32 | What are the benefits of exclusive breast feeding? (multiple answer) Yes=1, No=2 | | | | | | | | | |  | |  |
|  | 1. Beneficial for baby’s healthy growth | | | | | | | | | | \|__\| | |  |
|  | 1. Protect baby from diseases by improving resistence | | | | | | | | | | \|__\| | |  |
|  | 1. Work as contraceptive | | | | | | | | | | \|__\| | |  |
|  | 1. Baby becomes intelligent/brilliant | | | | | | | | | | \|__\| | |  |
|  | 1. Prevents potential stomach infection from other food | | | | | | | | | | \|__\| | |  |
|  | 1. Baby’s health becomes good | | | | | | | | | | \|__\| | |  |
|  | 1. I don’t know | | | | | | | | | | \|__\| | |  |
|  | 1. Others (specify) ....................................................... | | | | | | | | | | \|__\| | |  |
| 4.33 | How will you know if a baby is getting enough milk from breast feeding? (multiple answer) Yes=1, No=2 | | | | | | | | | |  | |  |
|  | 1. Baby will urinate for at least 6 times a day | | | | | | | | | | \|__\| | |  |
|  | 1. Baby will sleep properly | | | | | | | | | | \|__\| | |  |
|  | 1. Baby’s weight will increase | | | | | | | | | | \|__\| | |  |
|  | 1. Breasts will not be sore/engorged | | | | | | | | | | \|__\| | |  |
|  | 1. Baby itself will not want to feed more | | | | | | | | | | \|__\| | |  |
|  | 1. I don’t know | | | | | | | | | | \|__\| | |  |
|  | 1. Others (specify) ....................................................... | | | | | | | | | | \|__\| | |  |
| 4.34 | What is the duration after birth a baby should be started breast feeding? | | | | | | | | | | \|__\| | |  |
|  | 1. Within 1 hour after birth | | | | 1 | | | | | |  |  |  |
|  | 1. Within 2-6 hours after birth | | | | 2 | | | | | |  |  |  |
|  | 1. Within 7-12 hours after birth | | | | 3 | | | | | |  |  |  |
|  | 1. Within 13-24 hours after birth | | | | 4 | | | | | |  |  |  |
|  | 1. Within 1-3 days after birth | | | | 5 | | | | | |  |  |  |
|  | 1. Within 4-6 days after birth | | | | 6 | | | | | |  |  |  |
|  | 1. Within 7 days or more after birth | | | | 7 | | | | | |  |  |  |
|  | 1. I don’t know | | | | 9 | | | | | |  |  |  |
| 4.35 | What did you feed your baby for the first time after birth? | | | | | | | | | | \|__\| | | If answer is code 1 skip to 4.38 |
|  | 1. Colostrums | | | | 1 | | | | | |  |  |  |
|  | 1. breast milk without feeding the colostrums | | | | 2 | | | | | |  |  |  |
|  | 1. Bottle milk/Formula | | | | 3 | | | | | |  |  |  |
|  | 1. Sugar water | | | | 4 | | | | | |  |  |  |
|  | 1. Honey/fruit juice | | | | 5 | | | | | |  |  |  |
|  | 1. Others (specify) ....................................................... | | | | 6 | | | | | |  |  |  |
| 4.36 | Did you feed colostrums to your baby? | | | | | | | | | | \|__\| | | If answer is code 1 skip to 4.38 |
|  | 1. Yes | | | | 1 | | | | | |  |  |  |
|  | 1. No | | | | 2 | | | | | |  |  |  |
| 4.37 | Why didn’t you feed colostrums to your baby? (multiple answer) Yes=1, No=2 | | | | | | | | | |  | |  |
|  | 1. baby was sick | | | | | | | | | | \|__\| | |  |
|  | 1. Mother was sick | | | | | | | | | | \|__\| | |  |
|  | 1. Colostrum makes baby sick | | | | | | | | | | \|__\| | |  |
|  | 1. Colostrum makes baby fat | | | | | | | | | | \|__\| | |  |
|  | 1. Colostrum/breast milk was coming out | | | | | | | | | | \|__\| | |  |
|  | 1. I don’t know | | | | | | | | | | \|__\| | |  |
|  | 1. Others (specify) ...................................................... | | | | | | | | | | \|__\| | |  |
| 4.38 | Did you breast feed your newborn? | | | | | | | | | | \|__\| | | If answer is code 2 skip to 4.42 |
|  | 1. Yes | | | | | | 1 | | | |  |  |  |
|  | 1. No | | | | | | 2 | | | |  |  |  |
| 4.39 | What is the duration after birth you started breast feeding to your newborn? | | | | | | | | | \|__\| | | |  |
|  | 1. Within 1 hour after birth | | | | | 1 | | | |  |  |  |  |
|  | 1. Within 2-6 hours after birth | | | | | 2 | | | |  |  |  |  |
|  | 1. Within 7-12 hours after birth | | | | | 3 | | | |  |  |  |  |
|  | 1. Within 13-24 hours after birth | | | | | 4 | | | |  |  |  |  |
|  | 1. Within 1-3 days after birth | | | | | 5 | | | |  |  |  |  |
|  | 1. Within 4-6 days after birth | | | | | 6 | | | |  |  |  |  |
|  | 1. Within 7 days or more after birth | | | | | 7 | | | |  |  |  |  |
|  | 1. I don’t remember | | | | | 9 | | | |  |  |  |  |
| 4.40 | How long did you exclusively breast fed your baby without any other liquid supplements? (If less than 1 month write 00) | | Upto ................. months after births | | | | | | | \|__\|\|__\| | | |  |
| 4.41 | Are you currently breastfeeding to your baby? | | | | | | | | | \|__\| | | |  |
|  | 1. Yes | | | | | 1 | | | |  |  |  |  |
|  | 1. No | | | | | 2 | | | |  |  |  |  |
| 4.42 | At what age in months a baby should be started supplemental solid food? | | | | | | | | | \|__\|\|__\| | | |  |
|  | 1. ------------ months age | | | | |  | | | |  |  |  |  |
|  | 1. I don’t know | | | | | 99 | | | |  |  |  |  |
| 4.43 | Did you start feeding your child supplemental solid food? | | | | | | | | | \|__\| | | | If answer is code 2 skip to 4.46 |
|  | 1. Yes | | | | | 1 | | | |  |  |  |  |
|  | 1. No | | | | | 2 | | | |  |  |  |  |
| 4.44 | At what age in months you started to feed your child with supplemental solid food | | | | | | | | | \|__\|\|__\| | | |  |
|  | 1. ------------ months age | | | | |  | | | |  |  |  |  |
|  | 1. I don’t remember | | | | | 99 | | | |  |  |  |  |
| 4.45 | What are the type of supplemental solid food did you start to feed your baby?  (multiple answer) Yes=1, No=2 | | | | | | | | |  | | |  |
|  | 1. Khichuri/rice with pulses | | | | | | | | | \|__\| | | |  |
|  | 1. Rice | | | | | | | | | \|__\| | | |  |
|  | 1. Fruits | | | | | | | | | \|__\| | | |  |
|  | 1. Vegetables | | | | | | | | | \|__\| | | |  |
|  | 1. Egg | | | | | | | | | \|__\| | | |  |
|  | 1. Fish/meat | | | | | | | | | \|__\| | | |  |
|  | 1. Biscuit | | | | | | | | | \|__\| | | |  |
|  | 1. Semolina/Pearl sago | | | | | | | | | \|__\| | | |  |
|  | 1. Others (specify) ....................................................... | | | | | | | | | \|__\| | | |  |
| 4.46 | Vaccines against how many diseases should be given within 4 months of baby’s age? | | | | | | | | | \|__\|\|__\| | | |  |
|  | 1. ---------- number | | | | |  | | | |  |  |  |  |
|  | 1. I don’t know | | | | | 99 | | | |  |  |  |  |
| 4.47 | What are the diseases for which vaccines are given within 4 months of baby’s age? (multiple answer) Yes=1, No=2 | | | | | | | | |  | |  | |
|  | 1. Diphtheria | | | | | | | | | \|__\| | |  |  |
|  | 1. Whooping Cough | | | | | | | | | \|__\| | |  |  |
|  | 1. Tetanus | | | | | | | | | \|__\| | |  |  |
|  | 1. Polio | | | | | | | | | \|__\| | |  |  |
|  | 1. Jaundice/ Hepatitis | | | | | | | | | \|__\| | |  |  |
|  | 1. Meningitis | | | | | | | | | \|__\| | |  |  |
|  | 1. Measles | | | | | | | | | \|__\| | |  |  |
|  | 1. TB | | | | | | | | | \|__\| | |  |  |
|  | 1. I don’t know | | | | | | | | | \|__\| | |  |  |
|  | 1. Others (specify) ....................................................... | | | | | | | | | \|__\| | |  |  |
| 4.48 | At what age the baby should be given the first dose of polio vaccine? (For those who say the day of birth/just after birth, write ‘00’ days) | | | | | | | | | \|__\|\|__\| | |  | |
|  | 1. ................................ days after birth | | | | | |  | | |  |  |  |  |
|  | 1. I don’t know | | | | | | 99 | | |  |  |  |  |
| 4.49 | From where immunization for child is available? (multiple answer) Yes=1, No=2 | | | | | | | | |  | |  | |
|  | 1. Govt. hospital (Medical college hospital/district hospital/MCWC/upazila health complex) | | | | | | | | | \|__\| | |  |  |
|  | 1. Govt. health centre (UH&FWC/union sub centre) | | | | | | | | | \|__\| | |  |  |
|  | 1. Community clinic | | | | | | | | | \|__\| | |  |  |
|  | 1. Sattelite clinic | | | | | | | | | \|__\| | |  |  |
|  | 1. EPI campaign | | | | | | | | | \|__\| | |  |  |
|  | 1. Private hospital/clinic | | | | | | | | | \|__\| | |  |  |
|  | 1. NGO clinic | | | | | | | | | \|__\| | |  |  |
|  | 1. Private chamber of medical doctors | | | | | | | | | \|__\| | |  |  |
|  | 1. CSBA/FWV/nurse/paramedic’s home | | | | | | | | | \|__\| | |  |  |
|  | 1. Own home by health worker | | | | | | | | | \|__\| | |  |  |
|  | 1. I don’t know | | | | | | | | | \|__\| | |  |  |
|  | 1. Others (specify) ....................................................... | | | | | | | | | \|__\| | |  |  |
| 4.50 | Did you immunize your baby? | | | | |  | | | | \|__\| | | If answer is code 2 skip to Q 4.54 | |
|  | 1. Yes | | | | | 1 | | | |  |  |  |  |
|  | 1. No | | | | | 2 | | | |  |  |  |  |
| 4.51 | What was your baby’s age when you gave the first vaccine? For those who say the day of birth/just after birth, write ‘00’ days) | | | | | | | | | \|__\|\|__\| | |  | |
|  | 1. ................................ days after birth | | | | |  | | | |  |  |  |  |
|  | 1. I don’t remember | | | | | 99 | | | |  |  |  |  |
| 4.52 | What are the diseases against which you immunized your baby within four months age? (multiple answer) Yes=1, No=2 | | | | | | | | |  | |  | |
|  | 1. Diphtheria | | | | | | | | | \|__\| | |  |  |
|  | 1. Whooping Cough | | | | | | | | | \|__\| | |  |  |
|  | 1. Tetanus | | | | | | | | | \|__\| | |  |  |
|  | 1. Polio | | | | | | | | | \|__\| | |  |  |
|  | 1. Jaundice/ Hepatitis | | | | | | | | | \|__\| | |  |  |
|  | 1. Meningitis | | | | | | | | | \|__\| | |  |  |
|  | 1. Measles | | | | | | | | | \|__\| | |  |  |
|  | 1. TB | | | | | | | | | \|__\| | |  |  |
|  | 1. I don’t know | | | | | | | | | \|__\| | |  |  |
|  | 1. Others (specify) ....................................................... | | | | | | | | | \|__\| | |  |  |
| 4.53 | Where did you take your child for immunization? (multiple answer) Yes=1, No=2 | | | | | | | | |  | | Skip to 4.55 | |
|  | 1. Govt. hospital (Medical college hospital/district hospital/MCWC/upazila health complex) | | | | | | | | | \|__\| | |  |  |
|  | 1. Govt. health centre (UH&FWC/union sub centre) | | | | | | | | | \|__\| | |  |  |
|  | 1. Community clinic | | | | | | | | | \|__\| | |  |  |
|  | 1. Sattelite clinic | | | | | | | | | \|__\| | |  |  |
|  | 1. EPI campaign | | | | | | | | | \|__\| | |  |  |
|  | 1. Private hospital/clinic | | | | | | | | | \|__\| | |  |  |
|  | 1. NGO clinic | | | | | | | | | \|__\| | |  |  |
|  | 1. CSBA/FWV/nurse/paramedic’s home | | | | | | | | | \|__\| | |  |  |
|  | 1. Own home by health worker | | | | | | | | | \|__\| | |  |  |
|  | 1. Others (specify) ....................................................... | | | | | | | | | \|__\| | |  |  |
| 4.54 | Why didn’t you immunize your child? (multiple answer) Yes=1, No=2 | | | | | | | | |  | |  | |
|  | 1. I didn’t know from you to get vaccinated | | | | | | | | | \|__\| | |  |  |
|  | 1. I didn’t know vaccine was needed to be given | | | | | | | | | \|__\| | |  |  |
|  | 1. Vaccine centre was too far from home | | | | | | | | | \|__\| | |  |  |
|  | 1. There was no one to take care of my other children | | | | | | | | | \|__\| | |  |  |
|  | 1. Husband/other family members didn’t give permission | | | | | | | | | \|__\| | |  |  |
|  | 1. There was no one to take me to the vaccine centre | | | | | | | | | \|__\| | |  |  |
|  | 1. Due to lack of money | | | | | | | | | \|__\| | |  |  |
|  | 1. My baby was sick | | | | | | | | | \|__\| | |  |  |
|  | 1. I was busy in other work | | | | | | | | | \|__\| | |  |  |
|  | 1. Vaccine will make my baby ill | | | | | | | | | \|__\| | |  |  |
|  | 1. Vaccine will make my baby mentally disable | | | | | | | | | \|__\| | |  |  |
|  | 1. Vaccine was not neccessary | | | | | | | | | \|__\| | |  |  |
|  | 1. Others (specify) ....................................................... | | | | | | | | | \|__\| | |  |  |
| 4.55 | Can newborn babies have jaundice just after birth? | | | | | | | | | \|__\| | | If answer is code 2 or 9 skip to Q4.57 | |
|  | 1. Yes | | | 1 | | | | | |  |  |  |  |
|  | 1. No | | | 2 | | | | | |  |  |  |  |
|  | 1. I don’t know | | | 9 | | | | | |  |  |  |  |
| 4.56 | How many days after birth can a newborn baby have jaundice? | | | | | | | | | \|__\| | |  | |
|  | 1. Within 24 hours after birth | | | 1 | | | | | |  |  |  |  |
|  | 1. Within 3-10 days after birth | | | 2 | | | | | |  |  |  |  |
|  | 1. After 10 days of birth | | | 3 | | | | | |  |  |  |  |
|  | 1. I don’t know | | | 4 | | | | | |  |  |  |  |
|  | 1. Others (specify) ....................................................... | | | 5 | | | | | |  |  |  |  |
| 4.57 | For what colour of stool of newborn baby one should seek care from doctor?  (multiple answer) Yes=1, No=2 | | | | | | | | |  | |  | |
|  | 1. Yellow colour stool | | | | | | | | | \|__\| | |  |  |
|  | 1. Green colour stool | | | | | | | | | \|__\| | |  |  |
|  | 1. Red/blood mixed stool | | | | | | | | | \|__\| | |  |  |
|  | 1. White colour | | | | | | | | | \|__\| | |  |  |
|  | 1. I don’t know | | | | | | | | | \|__\| | |  |  |
|  | 1. Others (specify) ....................................................... | | | | | | | | | \|__\| | |  |  |
| 4.58 | What measures should be taken if a newborn baby has diarrhoea?  (multiple answer) Yes=1, No=2 | | | | | | | | |  | |  | |
|  | 1. Breast feeding should be frequent | | | | | | | | | \|__\| | |  |  |
|  | 1. Breast feeding should be less | | | | | | | | | \|__\| | |  |  |
|  | 1. Breast feeding should be stopped | | | | | | | | | \|__\| | |  |  |
|  | 1. Excess water should be fed | | | | | | | | | \|__\| | |  |  |
|  | 1. Zinc tablet should be given | | | | | | | | | \|__\| | |  |  |
|  | 1. Oral saline should be given | | | | | | | | | \|__\| | |  |  |
|  | 1. Doctor’s advice and care should be seeked | | | | | | | | | \|__\| | |  |  |
|  | 1. Coconut water should be given | | | | | | | | | \|__\| | |  |  |
|  | 1. I don’t know | | | | | | | | | \|__\| | |  |  |
|  | 1. Others (specify) ....................................................... | | | | | | | | | \|__\| | |  |  |
| 4.59 | When to seek care in case of diarrhoea of a baby?  (multiple answer) Yes=1, No=2 | | | | | | | | |  | |  | |
|  | 1. When diarrhea sustains more than one day | | | | | | | | | \|__\| | |  |  |
|  | 1. When baby is weak and faints | | | | | | | | | \|__\| | |  |  |
|  | 1. When baby is dehydrated | | | | | | | | | \|__\| | |  |  |
|  | 1. When baby vomits repeatedly | | | | | | | | | \|__\| | |  |  |
|  | 1. When baby’s passes frequent liquid stool | | | | | | | | | \|__\| | |  |  |
|  | 1. I don’t know | | | | | | | | | \|__\| | |  |  |
|  | 1. Others (specify) ....................................................... | | | | | | | | | \|__\| | |  |  |
| 4.60 | What are the signs of a healthy baby?  (multiple answer) Yes=1, No=2 | | | | | | | | |  | |  | |
|  | 1. baby is happy and playful | | | | | | | | | \|__\| | |  |  |
|  | 1. Baby gaining weight properly | | | | | | | | | \|__\| | |  |  |
|  | 1. Baby communicates and respond to sound | | | | | | | | | \|__\| | |  |  |
|  | 1. Baby’s stool is yellow and firm | | | | | | | | | \|__\| | |  |  |
|  | 1. Baby will look beautiful | | | | | | | | | \|__\| | |  |  |
|  | 1. I don’t know | | | | | | | | | \|__\| | |  |  |
|  | 1. Others (specify) ....................................................... | | | | | | | | | \|__\| | |  |  |
| 4.61 | What are the danger signs/health complications a newborn baby may have? (multiple answer) Yes=1, No=2 | | | | | | | | |  | |  | |
|  | 1. Cough and cold | | | | | | | | | \|__\| | |  |  |
|  | 1. Breathing difficulties | | | | | | | | | \|__\| | |  |  |
|  | 1. High Fever | | | | | | | | | \|__\| | |  |  |
|  | 1. Severe Diarrhea | | | | | | | | | \|__\| | |  |  |
|  | 1. Inability to breast feed | | | | | | | | | \|__\| | |  |  |
|  | 1. Fainting | | | | | | | | | \|__\| | |  |  |
|  | 1. Convulsion | | | | | | | | | \|__\| | |  |  |
|  | 1. Jaundice | | | | | | | | | \|__\| | |  |  |
|  | 1. Frequent vomitting | | | | | | | | | \|__\| | |  |  |
|  | 1. Pustules in the body | | | | | | | | | \|__\| | |  |  |
|  | 1. Cord infection | | | | | | | | | \|__\| | |  |  |
|  | 1. Coldness of skin | | | | | | | | | \|__\| | |  |  |
|  | 1. I don’t know | | | | | | | | | \|__\| | |  |  |
|  | 1. Others (specify) ....................................................... | | | | | | | | | \|__\| | |  |  |
| 4.62 | Do you know from where to seek care if newborn complication/danger signs arise?  (multiple answer) Yes=1, No=2 | | | | | | | | |  | |  | |
|  | 1. Govt. hospital (Medical college hospital/district hospital/MCWC/upazila health complex) | | | | | | | | | \|__\| | |  |  |
|  | 1. Govt. health centre (UH&FWC/union sub centre) | | | | | | | | | \|__\| | |  |  |
|  | 1. Community clinic | | | | | | | | | \|__\| | |  |  |
|  | 1. Sattelite clinic | | | | | | | | | \|__\| | |  |  |
|  | 1. Private hospital/clinic | | | | | | | | | \|__\| | |  |  |
|  | 1. NGO clinic | | | | | | | | | \|__\| | |  |  |
|  | 1. Private chamber of medical doctors | | | | | | | | | \|__\| | |  |  |
|  | 1. CSBA/FWV/nurse/paramedic’s home | | | | | | | | | \|__\| | |  |  |
|  | 1. Own home by skilled/trained provider | | | | | | | | | \|__\| | |  |  |
|  | 1. Own home by NGO worker | | | | | | | | | \|__\| | |  |  |
|  | 1. TTBA/TBA’s house | | | | | | | | | \|__\| | |  |  |
|  | 1. Village doctor’s house | | | | | | | | | \|__\| | |  |  |
|  | 1. Homeo doctor’s house | | | | | | | | | \|__\| | |  |  |
|  | 1. Traditional healer’s house | | | | | | | | | \|__\| | |  |  |
|  | 1. Pharmacy | | | | | | | | | \|__\| | |  |  |
|  | 1. I don’t know | | | | | | | | | \|__\| | |  |  |
|  | 1. Others (specify) ....................................................... | | | | | | | | | \|__\| | |  |  |
| 4.63 | | Did your newborn baby had any complication/danger signs? | | | | | | | | \|__\| | | If answer is code 2 skip to Q 4.68 | |
|  |  | 1. Yes | | 1 | | | | | |  |  |  |  |
|  |  | 1. No | | 2 | | | | | |  |  |  |  |
| 4.64 | | What are the danger signs/complications your newborn baby had?  (multiple answer) Yes=1, No=2 | | | | | | | |  | |  | |
|  |  | 1. Cough and cold | | | | | | | | \|__\| | |  |  |
|  |  | 1. Breathing difficulties | | | | | | | | \|__\| | |  |  |
|  |  | 1. High Fever | | | | | | | | \|__\| | |  |  |
|  |  | 1. Severe Diarrhea | | | | | | | | \|__\| | |  |  |
|  |  | 1. Inability to breast feed | | | | | | | | \|__\| | |  |  |
|  |  | 1. Fainting | | | | | | | | \|__\| | |  |  |
|  |  | 1. Convulsion | | | | | | | | \|__\| | |  |  |
|  |  | 1. Jaundice | | | | | | | | \|__\| | |  |  |
|  |  | 1. Frequent vomitting | | | | | | | | \|__\| | |  |  |
|  |  | 1. Pustules in the skin | | | | | | | | \|__\| | |  |  |
|  |  | 1. Cord infection | | | | | | | | \|__\| | |  |  |
|  |  | 1. Coldness of skin | | | | | | | | \|__\| | |  |  |
|  |  | 1. Others (specify) ....................................................... | | | | | | | | \|__\| | |  |  |
| 4.65 | | Did you seek care for your newborn for the complications/danger signs? | | | | | | | | \|__\| | | If answer is code 2 Skip to Q4.67 | |
|  |  | 1. Yes | | | | 1 | | | |  |  |  |  |
|  |  | 1. No | | | | 2 | | | |  |  |  |  |
| 4.66 | | From where/whom did you seek care when your baby had complications/danger signs?  (multiple answer) Yes=1, No=2 | | | | | | | |  | | Go to 4.68 | |
|  |  | 1. Govt. hospital (Medical college hospital/district hospital/MCWC/upazila health complex) | | | | | | | | \|__\| | |  |  |
|  |  | 1. Govt. health centre (UH&FWC/union sub centre) | | | | | | | | \|__\| | |  |  |
|  |  | 1. Community clinic | | | | | | | | \|__\| | |  |  |
|  |  | 1. Sattelite clinic | | | | | | | | \|__\| | |  |  |
|  |  | 1. Private hospital/clinic | | | | | | | | \|__\| | |  |  |
|  |  | 1. NGO clinic | | | | | | | | \|__\| | |  |  |
|  |  | 1. Private chamber of medical doctors | | | | | | | | \|__\| | |  |  |
|  |  | 1. CSBA/FWV/nurse/paramedic’s home | | | | | | | | \|__\| | |  |  |
|  |  | 1. Own home by skilled/trained provider | | | | | | | | \|__\| | |  |  |
|  |  | 1. Own home by NGO worker | | | | | | | | \|__\| | |  |  |
|  |  | 1. TTBA/TBA’s house | | | | | | | | \|__\| | |  |  |
|  |  | 1. Village doctor’s house | | | | | | | | \|__\| | |  |  |
|  |  | 1. Homeo doctor’s house | | | | | | | | \|__\| | |  |  |
|  |  | 1. Traditional healer’s house | | | | | | | | \|__\| | |  |  |
|  |  | 1. Pharmacy | | | | | | | | \|__\| | |  |  |
|  |  | 1. Others (specify) ....................................................... | | | | | | | | \|__\| | |  |  |
| 4.67 | | Why didn’t you seek care for your newborn complication/danger signs?  (multiple answer) Yes=1, No=2 | | | | | | | |  | |  | |
|  |  | 1. I didn’t know from where to seek care | | | | | | | | \|__\| | |  |  |
|  |  | 1. I couldn’t afford the cost of hospital expenses | | | | | | | | \|__\| | |  |  |
|  |  | 1. Doctor is not available all the time in hospital | | | | | | | | \|__\| | |  |  |
|  |  | 1. There is possibility of wrong treatment in hospital | | | | | | | | \|__\| | |  |  |
|  |  | 1. Hopital was too far from home | | | | | | | | \|__\| | |  |  |
|  |  | 1. There was no one to take care of my other children | | | | | | | | \|__\| | |  |  |
|  |  | 1. Husband/other family members didn’t give permission | | | | | | | | \|__\| | |  |  |
|  |  | 1. There was no one to take me to the hospital | | | | | | | | \|__\| | |  |  |
|  |  | 1. I didn’t think it was necessary | | | | | | | | \|__\| | |  |  |
|  |  | 1. Others (specify) | | | | | | | | \|__\| | |  |  |
| 4.68 | | At what age a baby start to sit upright by itself? | | | | | | | | \|__\|\|__\| | |  | |
|  |  | 1. ........................ months age | | | |  | | | |  |  |  |  |
|  |  | 1. I Don’t know | | | | 99 | | | |  |  |  |  |
| 4.69 | | At what age the baby can hold her/his head steady? | | | | | | | | \|__\|\|__\| | |  | |
|  |  | 1. ........................ months age | | | |  | | | |  |  |  |  |
|  |  | 1. I Don’t know | | | | 99 | | | |  |  |  |  |
| 4.70 | | Did you communicate/talk with your baby since birth? | | | | | | | | \|__\| | |  | |
|  |  | 1. Yes | | | | 1 | | | |  |  |  |  |
|  |  | 1. No | | | | 2 | | | |  |  |  |  |
| 4.71 | | Did you play with your baby using colourful toys since birth? | | | | | | | | \|__\| | |  | |
|  |  | 1. Yes | | | | 1 | | | |  |  |  |  |
|  |  | 1. No | | | | 2 | | | |  |  |  |  |
| 4.72 | | What is the necessity of new mothers to eat extra food after delivery?  (multiple answer) Yes=1, No=2 | | | | | | | |  | |  | |
|  |  | 1. For own good health | | | | | | | | \|__\| | |  |  |
|  |  | 1. For passing nutrition to baby through breast milk | | | | | | | | \|__\| | |  |  |
|  |  | 1. To improve resistence against diseases¨ | | | | | | | | \|__\| | |  |  |
|  |  | 1. To produce more breast milk | | | | | | | | \|__\| | |  |  |
|  |  | 1. I don’t know | | | | | | | | \|__\| | |  |  |
|  |  | 1. Others (specify) ....................................................... | | | | | | | | \|__\| | |  |  |
| 4.73 | | During last postnatal period, how much food you ate? | | | | | | | | \|__\| | |  | |
|  |  | 1. More than usual | | 1 | | | | | |  |  |  |  |
|  |  | 1. Same like usual | | 2 | | | | | |  |  |  |  |
|  |  | 1. Less than usual | | 3 | | | | | |  |  |  |  |
| 4.74 | | During last postnatal period, what are the type of nutritious food you ate?  (multiple answer) Yes=1, No=2 | | | | | | | |  | |  | |
|  |  | 1. Vegetable | | | | | | | | \|__\| | |  |  |
|  |  | 1. Fish/meat | | | | | | | | \|__\| | |  |  |
|  |  | 1. Milk | | | | | | | | \|__\| | |  |  |
|  |  | 1. Egg | | | | | | | | \|__\| | |  |  |
|  |  | 1. Fruits | | | | | | | | \|__\| | |  |  |
|  |  | 1. Pulses/beans | | | | | | | | \|__\| | |  |  |
|  |  | 1. Lot of water | | | | | | | | \|__\| | |  |  |
|  |  | 1. Others (specify) ....................................................... | | | | | | | | \|__\| | |  |  |
| 4.75 | | During last postnatal period, did you take Vitamin A capsule? | | | | | | | | \|__\| | | If answer is code 2 or 9 skip to Q 4.77 | |
|  |  | 1. Yes | | | | 1 | | | |  |  |  |  |
|  |  | 1. No | | | | 2 | | | |  |  |  |  |
|  |  | 1. I don’t know/I don’t remember | | | | 9 | | | |  |  |  |  |
| 4.76 | | Within how many days after delivery you took vitamin A capsule? | | | | | | | | \|__\|\|__\| | |  | |
|  |  | 1. …………………… days after birth | | | |  | | | |  |  |  |  |
|  |  | 1. I don’t know/I don’t remember | | | | 99 | | | |  |  |  |  |
| 4.77 | | During last postnatal period, did you take Iron/Folic Acid tablet? | | | | | | | | \|__\| | | If answer is code 2 or 9 skip to Q 4.79 | |
|  |  | 1. Yes | | | | 1 | | | |  |  |  |  |
|  |  | 1. No | | | | 2 | | | |  |  |  |  |
|  |  | 1. I don’t know/I don’t remember | | | | 9 | | | |  |  |  |  |
| 4.78 | | During last postnatal period, why did you take Iron/Folic acid tablet?  (multiple answer) Yes=1, No=2 | | | | | | | |  | |  | |
|  |  | 1. For anaemia correction | | | | | | | | \|__\| | |  |  |
|  |  | 1. For physical weakness | | | | | | | | \|__\| | |  |  |
|  |  | 1. Doctor prescribed it | | | | | | | | \|__\| | |  |  |
|  |  | 1. I don’t know | | | | | | | | \|__\| | |  |  |
|  |  | 1. Others (specify) ....................................................... | | | | | | | | \|__\| | |  |  |
| 4.79 | | During last postnatal period, did you take Calcium tablet? | | | | | | | | \|__\| | | If answer is code 2 or 9 skip to Q 4.81 | |
|  |  | 1. Yes | | | | 1 | | | |  |  |  |  |
|  |  | 1. No | | | | 2 | | | |  |  |  |  |
|  |  | 1. I don’t know/I don’t remember | | | | 9 | | | |  |  |  |  |
| 4.80 | | During last postnatal period, why did you take Calcium acid tablet?  (multiple answer) Yes=1, No=2 | | | | | | | |  | |  | |
|  |  | 1. To strengthen her bones | | | | | | | | \|__\| | |  |  |
|  |  | 1. To reduce her physical weakness | | | | | | | | \|__\| | |  |  |
|  |  | 1. Doctor prescribed it | | | | | | | | \|__\| | |  |  |
|  |  | 1. Baby will get more breast milk | | | | | | | | \|__\| | |  |  |
|  |  | 1. I don’t know | | | | | | | | \|__\| | |  |  |
|  |  | 1. Others (specify) ....................................................... | | | | | | | | \|__\| | |  |  |
| 4.81 | | How many years of birth spacing a woman should take after delivery? | | | | | | | | \|__\|\|__\| | |  | |
|  |  | 1. ………………… years | |  | | | | | |  |  |  |  |
|  |  | 1. I don’t know | | 99 | | | | | |  |  |  |  |
| 4.82 | | What are the long term methods of contraception?  (multiple answer) Yes=1, No=2 | | | | | | | |  | |  | |
|  |  | 1. Injection | | | | | | | | \|__\| | |  |  |
|  |  | 1. Copper T/Intrauterine device | | | | | | | | \|__\| | |  |  |
|  |  | 1. Implant/Norplant | | | | | | | | \|__\| | |  |  |
|  |  | 1. I don’t know | | | | | | | | \|__\| | |  |  |
| 4.83 | | After the last delivery, did you take any kind of contraceptives? | |  | | | | | | \|__\| | |  | |
|  |  | 1. Yes | | 1 | | | | | |  |  |  |  |
|  |  | 1. No | | 2 | | | | | |  |  |  |  |

**SECTION 5: ON SERVICE AND INTENT OF SERVICE**

1. **TO USERS OF APONJON**

| **No** | **Questions and Filters** | **Coding** | **Responce** | **Skip** |
| --- | --- | --- | --- | --- |
| 5.1 | Will you be continuing as a subscriber of Aponjon/MAMA mHealth programme? | | \|__\| | If answer is code 1, skip to Q 5.3 |
|  | 1. Yes | 1 |  |  |
|  | 1. No | 2 |  |  |
| 5.2 | Why you don’t want to continue Aponjon/MAMA mHealth programme?  (multiple answer) Yes=1, No=2 | |  |  |
|  | 1. I can’t afford the cost of messages of Aponjon | | \|__\| |  |
|  | 1. The messages are not useful | | \|__\| |  |
|  | 1. There are many other sources of messages available | | \|__\| |  |
|  | 1. Family members do not want me to continue | | \|__\| |  |
|  | 1. Aponjon cannot provide any health care | | \|__\| |  |
|  | 1. Aponjon can not refer me where my health care can be ensured | | \|__\| |  |
|  | 1. Others (specify) ...................................................... | | \|__\| |  |
| 5.3 | Did you suggest anybody to take the Aponjon/MAMA mHealth programme? | | \|__\| | If answer is code 1, skip to Q 5.5 |
|  | 1. Yes | 1 |  |  |
|  | 1. No | 2 |  |  |
| 5.4 | Whom did you suggest to take the Aponjon/MAMA mHealth programme?  (multiple answer) Yes=1, No=2 | |  |  |
|  | 1. Family members | | \|__\| |  |
|  | 1. Friends/Relatives | | \|__\| |  |
|  | 1. Neighbours | | \|__\| |  |
|  | 1. Others (specify) ...................................................... | | \|__\| |  |
| 5.5 | Did you discuss about Aponjon/MAMA mHealth programme with any pregnant/new mother | | \|__\| | If answer is code 2 end the interview |
|  | 1. Yes | 1 |  |  |
|  | 1. No | 2 |  |  |
| 5.6 | Which messages of Aponjon/MAMA mHealth programme did you discuss with pregnant/new mother?  (multiple answer) Yes=1, No=2 | |  |  |
|  | 1. Antenatal/delivery care related information | | \|__\| |  |
|  | 1. Symptoms of pregnancy/delivery complications | | \|__\| |  |
|  | 1. Necessity of birth planning | | \|__\| |  |
|  | 1. Information on immunization during pregnancy | | \|__\| |  |
|  | 1. Postnatal care related information | | \|__\| |  |
|  | 1. Newborn care related information | | \|__\| |  |
|  | 1. Information on mental development of child | | \|__\| |  |
|  | 1. Prevention and management of diarrhoea of child | | \|__\| |  |
|  | 1. Sympotoms of newborn health complications | | \|__\| |  |
|  | 1. Source of maternal and newborn healthcare | | \|__\| |  |
|  | 1. Benefit of breast feeding | | \|__\| |  |
|  | 1. Information on maternal nutrition | | \|__\| |  |
|  | 1. Information on child nutrition | | \|__\| |  |
|  | 1. Information on immunization of child | | \|__\| |  |
|  | 1. Family planning advice | | \|__\| |  |
|  | 1. Hygeine advice | | \|__\| |  |
|  | 1. I don’t know | | \|__\| |  |
|  | 1. Others (specify) ....................................................... | | \|__\| |  |
| 5.7 | Did anyone subscribe to Aponjon/MAMA mHealth programme being influenced by by you? | | \|__\| | If answer is code 2 or 9 end the interview |
|  | 1. Yes | 1 |  |  |
|  | 1. No | 2 |  |  |
|  | 1. I don’t know | 9 |  |  |
| 5.8 | How many people subscribed to Aponjon/MAMA mHealth programme being influenced by you? | | \|__\|\|__\| | If answer is code 99 end the interview |
|  | 1. -------- number |  |  |  |
|  | 1. I don’t know | 99 |  |  |
| 5.9 | Who subscribed to Aponjon/MAMA mHealth programme being influenced by by you?  (multiple answer) Yes=1, No=2 | |  | End of interview |
|  | 1. Family members | | \|__\| |  |
|  | 1. Friends | | \|__\| |  |
|  | 1. Relatives | | \|__\| |  |
|  | 1. Neighbours | | \|__\| |  |
|  | 1. Others (specify) ....................................................... | | \|__\| |  |

**The non-users who never heard about Aponjon/MAMA mHealth programme, explain the programme to her and then go to section 5B**

1. **TO NON USERS OF APONJON**

| **No** | **Questions and Filters** | **Coding** | **Responce** | **Skip** |
| --- | --- | --- | --- | --- |
| 5.10 | Will you be interested in receiving health messages from Aponjon/MAMA mHealth programme? | | \|__\| | If answer is code 1 skip to Q5.12 |
|  | 1. Yes | 1 |  |  |
|  | 1. No | 2 |  |  |
| 5.11 | Why you are not interested to receive health messages from Aponjon/MAMA mHealth programme?  (multiple answer) Yes=1, No=2 | |  | End of interview |
|  | 1. There are many other sources of health messages available | | \|__\| |  |
|  | 1. It cost money to get Aponjon health messages | | \|__\| |  |
|  | 1. I do not have the access to mobile phone | | \|__\| |  |
|  | 1. Others (specify) ...................................................... | | \|__\| |  |
| 5.12 | What sort of messages would you like to receive on maternal and newborn health from Aponjon/MAMA mHealth programme?  (multiple answer) Yes=1, No=2 | |  |  |
|  | 1. Antenatal/delivery care related information | | \|__\| |  |
|  | 1. Symptoms of pregnancy/delivery complications | | \|__\| |  |
|  | 1. Necessity of birth planning | | \|__\| |  |
|  | 1. Information on immunization during pregnancy | | \|__\| |  |
|  | 1. Postnatal care related information | | \|__\| |  |
|  | 1. Newborn care related information | | \|__\| |  |
|  | 1. Information on mental development of child | | \|__\| |  |
|  | 1. Prevention and management of diarrhoea of child | | \|__\| |  |
|  | 1. Sympotoms of newborn health complications | | \|__\| |  |
|  | 1. Source of maternal and newborn healthcare | | \|__\| |  |
|  | 1. Benefit of breast feeding | | \|__\| |  |
|  | 1. Information on maternal nutrition | | \|__\| |  |
|  | 1. Information on child nutrition | | \|__\| |  |
|  | 1. Information on immunization of child | | \|__\| |  |
|  | 1. Family planning advice | | \|__\| |  |
|  | 1. Hygeine advice | | \|__\| |  |
|  | 1. I don’t know | | \|__\| |  |
|  | 1. Others (specify) ....................................................... | | \|__\| |  |
| 5.13 | Will you be interested to pay any money to receive health messages from Aponjon/MAMA mHealth programme? | | \|__\| | If answer is code 2 end of interview |
|  | 1. Yes | 1 |  |  |
|  | 1. No | 2 |  |  |
| 5.14 | How much will you be interested to pay each health message from Aponjon/MAMA mHealth programme? | |  | End of interview |
|  | ......................................................................... Taka | |  |  |

**time of end of interview**: |___|___|___|___| [Railway time] Date: |___|___| |___|___| |___|___|

Name of interviewer ----------------------------------- Code No. |___|___| Date |___|___| |___|___| |___|___|

Name of Supervisor ------------------------------------ Code No. |___|___| Date |___|___| |___|___| |___|___|

Name of QCO ------------------------------------------ Code No. |___|___| Date |___|___| |___|___| |___|___|

Name of data entry operator --------------------------- Code No. |___|___|Date |___|___| |___|___| |___|___|
